# Correlation Between Genetic Abnormalities in Induced Pluripotent Stem Cell-Derivatives and Abnormal Tissue Formation in Tumorigenicity Tests

**DOI:** 10.1093/stcltm/szac014

**Published:** 2022-04-21

**Authors:** Takako Yamamoto, Yoji Sato, Satoshi Yasuda, Masayuki Shikamura, Takashi Tamura, Chiemi Takenaka, Naoko Takasu, Masaki Nomura, Hiromi Dohi, Masayo Takahashi, Michiko Mandai, Yonehiro Kanemura, Masaya Nakamura, Hideyuki Okano, Shin Kawamata

**Affiliations:** 1 R&D Center for Cell Therapy, Foundation for Biomedical Research and Innovation, Kobe, Japan; 2 Division of Cell-Based Therapeutic Products, National Institute of Health Sciences, Kawasaki, Japan; 3 CiRA Foundation, Kyoto, Japan; 4 Riken BDR, Kobe, Japan; 5 Vison Care Inc., Kobe, Japan; 6 Department of Biomedical Research and Innovation, Institute for Clinical Research, National Hospital Organization Osaka National Hospital, Osaka, Japan; 7 Department of Orthopedic Surgery, Keio University School of Medicine, Tokyo, Japan; 8 Department of Physiology, Keio University School of Medicine, Tokyo, Japan

**Keywords:** whole-genome sequencing, tumorigenicity test, Census database, Shibata List, single-nucleotide variants, copy number variants, variation of allele frequency

## Abstract

Cell therapy using induced pluripotent stem cell (iPSC) derivatives may result in abnormal tissue generation because the cells undergo numerous cycles of mitosis before clinical application, potentially increasing the accumulation of genetic abnormalities. Therefore, genetic tests may predict abnormal tissue formation after transplantation. Here, we administered iPSC derivatives with or without single-nucleotide variants (SNVs) and deletions in cancer-related genes with various genomic copy number variant (CNV) profiles into immunodeficient mice and examined the relationships between mutations and abnormal tissue formation after transplantation. No positive correlations were found between the presence of SNVs/deletions and the formation of abnormal tissues; the overall predictivity was 29%. However, a copy number higher than 3 was correlated, with an overall predictivity of 86%. Furthermore, we found CNV hotspots at 14q32.33 and 17q12 loci. Thus, CNV analysis may predict abnormal tissue formation after transplantation of iPSC derivatives and reduce the number of tumorigenicity tests.

## Graphical Abstract

**Graphical Abstract:**
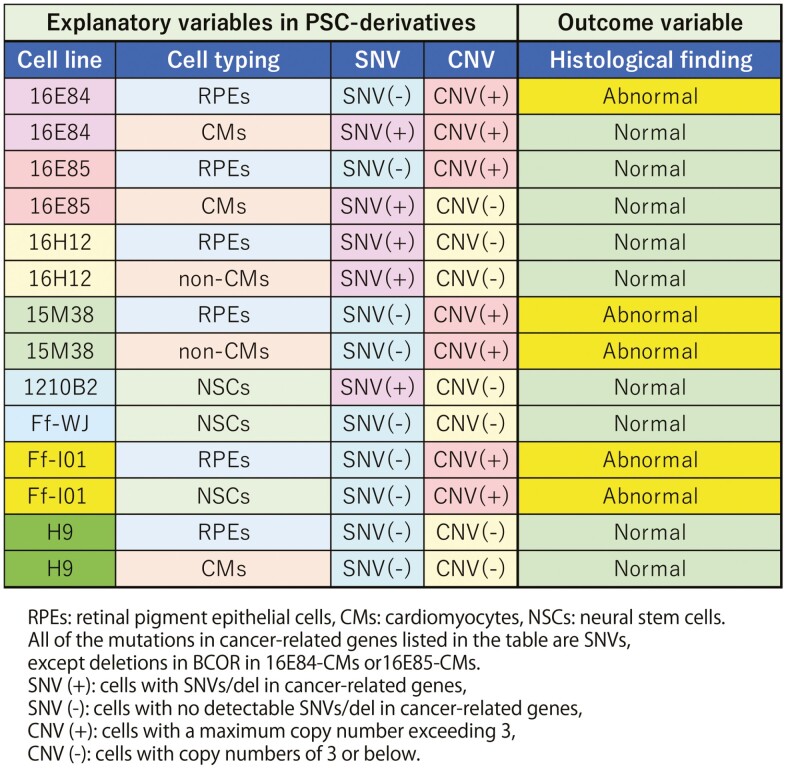

**Significance Statement**

We administered iPSC derivatives with or without single-nucleotide variants (SNVs) and with various genomic copy number variant (CNV) profiles into immunodeficient mice and examined the relationships between mutations and abnormal tissue formation after transplantation. No positive correlations were found between the presence of SNVs and the formation of abnormal tissues. However, a copy number higher than 3 was correlated. Furthermore, we found CNV hotspots at 14q32.33 and 17q12 loci. Thus, CNV analysis may predict abnormal tissue formation after transplantation of iPSC derivatives.

## Introduction

Cell therapy has recently been achieved using induced pluripotent stem cell (iPSC) derivatives.^1-4^ However, with this approach, tumorigenic events may occur after transplantation. Genetic abnormalities and impurity with residual undifferentiated cells and transformed cells in iPSC derivatives may contribute to abnormal tissue formation after transplantation. Because the number of accumulated mitoses in iPSC derivatives during reprogramming, cloning, and maintenance of culture and differentiation is considerably high compared with that in somatic cells used for cell therapy, the chance of genetic abnormalities is also increased.^5,6^ Therefore, it is important to evaluate the genetic profiles of the cells prior to transplantation and examine whether detected genetic abnormalities could be predictive of abnormal tissue generation.

Most current information regarding cancer-related genetic abnormalities is found in the Catalogue of Somatic Mutation in Cancer (COSMIC; https://cancer.sanger.ac.uk/cosmic). The COSMIC database consists of 2 distinct Cancer Gene databases; the first is Cancer Gene Census (hereafter known as Census), a list of hundreds of genes generated from oncogenes reported in published cancer cases,^7^ and the second is a database of existing databases, including The Cancer Genome Atlas (https://www.cancer.gov/about-nci/organization/ccg/research/structural-genomics/tcga) and International Cancer Genome Consortium (https://dcc.icgc.org/). In addition to the COSMIC database, a guidance document published by the Japan Ministry of Health, Labor and Welfare recommends a comprehensive search for mutations of cancer-associated genes in Shibata’s List^8^ prior to the first-in-human clinical studies on iPSC derivatives, which are conducted under the Act on the Safety of Regenerative Medicine.^9^ However, cells having mutations in oncogene databases do not always generate tumor because our bodies consist of genetically mosaic cells with various mutations.^10^ Moreover, some individuals lead cancer-free lives for several decades after detection or acquisition of major cancer-driver gene mutations,^11^ and the mechanisms underlying this apparent oncogene tolerance in human cells have recently been evaluated.^12-14^ Genes expressed in cells exert their functions depending on the given microenvironment or designated developmental stage; therefore, cells with genetic abnormalities may not necessarily exert the same functions if the microenvironment or developmental stage is different from the original cancer. In fact, disseminated tumor cells enter a state of dormancy in secondary organs, and this state is strongly influenced by the organ microenvironment.^15-17^ Because we do not have sufficient accumulated knowledge to link genetic abnormalities in transplanted cells with the tissue phenotype after transplantation considering the effects of the microenvironment, tumorigenicity tests using the same methods as used in the clinical setting may be indispensable as definitive safety tests. Therefore, it would be beneficial to establish robust quality control (QC) tests to check candidate cell clones and reduce the need for tumorigenicity tests in animals, which require an observation period of 1-1.5 years.^18^

Accordingly, in this study, we transplanted iPSC derivatives with or without known genetic mutations in the Census database and/or Shibata’s list into immunodeficient (NOD/Shi-scid, IL-2Rγ-null [NOG]) mice^19^ to explore the correlations between the presence of reported genetic mutations in cancer-related genes and the formation of abnormal tissues after transplantation.

## Experimental Procedures

All experiments using human samples and animal studies were reviewed by the Institutional Review Board of the Foundation for Biomedical Research and Innovation (FBRI) and the committee for animal experiments of the FBRI.

### Study Design

The relationship between mutations in cancer-related genes or CNVs of iPSC derivatives and the formation of abnormal transplants, including tumors or off-target tissues, were examined by transplanting iPSC-RPEs, -CMs, or -NSCs of various CNVs with or without known genetic mutations, as determined by WGS, into 6-8 immune-deficient mice for respective derivatives. We then examined the histology of tissues transplanted via a subcutaneous (RPEs, CMs) or clinical route (NSCs).

### Cell Culture

iPSC clones included the following cells: 1210B2 (peripheral blood mononuclear cell [PBMC] origin), Ff-WJ14s01 (cord blood cell origin), and Ff-I01 (PBMC origin). iPSC clones with known genetic mutations included the following: 16E84 (PBMC origin, QHJI01s01 derivative), 16E85 (PBMC origin, QHJI01s01 derivative), 16H12 (PBMC origin, DRXT22 derivative), and 15M38 (cord blood cell origin, YZWJ24 derivative); all iPSC clones were obtained from CiRA-Foundation, Kyoto University. 16E84, 16E85, 16H12, and 15M38 carry known mutations in cancer-related genes, and their derivatives were used in this study as positive samples of final products with mutations in order to address the relationships between genetic mutations in cells and their histological outcomes after transplantation. These iPSC clones were established at CiRA, Kyoto University from donors after obtaining informed consent, and their use was approved by the Ethics Committee of the Faculty of Medicine, Kyoto University.

H9 ESCs (WiCells),^48^ which harbored no mutations in the Census database (http://cancer.sanger.ac.uk/census) or Shibata’s List (https://www.pmda.go.jp/files/000152599.pdf), were used as controls. These cells were seeded as single-cell suspensions at 1.3 × 10^4^ cells/well in a 6-well plate coated with iMatrix-511 (Matrixome Inc.) and cultured with StemFit AK02N (AJINOMOTO HEALTHY SUPPLY Co., Inc.). A ROCK inhibitor (Y-27632; Nacalai Tesque) was used only at the time of plating. The medium was changed every other day, and the cells were cultured in an incubator (PHCbi) at 37 °C in an atmosphere containing 5% CO_2_. Eight to 10 days after plating, the cells reached 80-90% confluence and were ready for passaging. TrypLE Select (TrypLE diluted 1:1 with 0.5 mM ethylenediaminetetraacetic acid/phosphate-buffered saline [PBS] (−)) for 4 minutes at 37 °C. Because the cells remained attached to iMatrix-511-coated plates, we aspirated the TrypLE Select and carefully washed the cells with PBS(−). The cells were scraped under StemFit AK02N and dissociated into single cells by pipetting the cells 10 times. Morphologies were observed by phase-contrast microscopy (IX81; Olympus). These cells were seeded as single-cell suspensions of iPSCs at 1 × 10^5^ cells/well in a 6-well plate (Corning) coated with recombinant human VTN-N (Thermo Fisher Scientific, Waltham, MA, USA) and cultured with Essential 8 Medium (Thermo Fisher Scientific). Y-27632 was used only at the time of plating to conduct embryoid body formation assays. The culture medium was changed every day, and the cells were cultured in an incubator (PHCbi) at 37 °C in an atmosphere containing 5% CO_2_. Three days after seeding, cells were harvested using TrypLE Select (Thermo Fisher Scientific) for single-cell passaging.

### RPE Differentiation

A modified RPE differentiation protocol was used in this experiment. Briefly, human iPSCs were cultured on gelatin-coated dishes in Glasgow’s MEM (GMEM; Thermo Fisher Scientific) with several supplements, including KnockOut Serum Replacement (Thermo Fisher Scientific). Signal inhibitors Y-27632 (Wako), SB43542 (Sigma, St. Louis, MO, USA), and CKI-7 (Sigma) were added to the GMEM. After the appearance of RPE-like colonies, cells were dissociated with Disperse (Godoshuzho, Japan), filtered with a 40-μm filter (BD) to remove debris, and cultured with RPE maintenance medium (DMEM+F12, l-glutamine, and B27) on MPC-coated dishes (Nunc) for 10 days (p1). The cells were then harvested with trypsin (Lonza), filtered with a 40-μm filter (BD) to remove debris, and cultured with RPE maintenance medium on iMatrix-511(Matrixome Inc., Japan)-coated dishes (p2) before transplantation.

### CM Differentiation

Differentiation into CMs was performed using a PSC Cardiomyocyte Differentiation Kit (Thermo Fisher Scientific) according to the manufacturer’s protocol. On differentiation day 12, hiPSC-derived cells were incubated with glucose and glutamine-free DMEM (Thermo Fisher Scientific) supplemented with 4 mM l-lactic acid (Sigma-Aldrich) and 0.1% bovine serum albumin (Thermo Fisher Scientific) for 3 days. On day 15, the medium was changed to RPMI with B27 plus insulin (Thermo Fisher Scientific). After metabolic selection on day 17, purified hiPSC-CMs were used for immunostaining with cardiac troponin T.

### NSC Differentiation

Human iPSCs were induced into NPCs using the dual SMAD inhibition method with dorsomorphin (FUJIFILM Wako Pure Chemical Corporation, Osaka, Japan) or LDN-193189 (Axon Medchem, Groningen, The Netherlands) plus SB431542 (Sigma-Aldrich) and propagated using the neurosphere method in DMEM/Ham’s F-12 (DMEM/F12; FUJIFILM Wako Pure Chemical Corporation) supplemented with epidermal growth factor (20 ng/mL; PeproTech, Rocky Hill, NJ, USA), fibroblast growth factor 2 (20 ng/mL; PeproTech), leukemia inhibitory factor (10 ng/mL; Millipore, Billerica, MA, USA), B27 supplement (B27, 2%; Thermo Fisher Scientific), and heparin (1/1000 dilution; AY Pharmaceuticals, Tokyo, Japan) as previously described.^18^

### Tumorigenicity Tests

For tumorigenicity tests, 1 × 10^6^ iPSC derivatives (RPEs, non RPEs, CMs, or non CMs) were transplanted subcutaneously into 6 NOG mice (NOD/ShiJic-scid, IL-2Rγ KO Jic; CLEA Japan) in each group. Additionally, 1 × 10^6^ iPSC-NPCs were injected into the striatum of 6 NOG mice. Cells were embedded in 100 μL Matrigel (BD Biosciences) followed by injection subcutaneously with a 1-mL syringe (TERMO) with a 26-G needle. Tumor formation was observed for up to 4-6 months. Alternatively, NPCs were injected into the striatum with a 1-mL syringe with a 26-G needle and observed for up to 4-6 months. At the end of the experiments, mice were sacrificed, and transplants were removed and fixed with 4% paraformaldehyde. Paraffin-embedded sections were stained with hematoxylin and eosin (HE) for histological observation by Sapporo General Pathology Laboratory (https://www.sgpl.co.jp/e_idx.html).

### HE Staining and Immunohistochemistry

Transplanted mouse brain tissues were fixed with 4% paraformaldehyde in PBS. Paraffin-embedded tissue sections were stained with HE. Then, the paraffin sections were deparaffinized with xylene and sequential 100%, 95%, 80%, 70% ethanol treatments for 5 minutes each. Antigen retrieval was performed with Target Retrieval Solution (cat. no. S1699; Dako) at 121 °C for 20 minutes, followed by suppression of endogenous peroxidase activity with 3% hydrogen peroxide solution in methanol at room temperature for 10 minutes. After blocking with 10% normal goat serum, deparaffinized sections were incubated at 4 °C overnight with antibodies targeting human Nestin (1:200; cat. no. 18741; IBL), human nuclei (1:1500; cat. no. MAB4383; Millipore), human Ki-67 (1:50; cat. no. M7240; Dako), STEM121 (1:1000; cat. no. Y40410; Takara Bio, Shiga, Japan), and STEM123 (1:1000; cat. no. Y40420; Takara Bio). Histofine Simple Stain MAX PO (M) (cat. no. 424131; Nichirei Biosciences Inc.) or Histofine Simple Stain MAX PO (R) (cat. no. 424141; Nichirei Biosciences Inc.) was used as a secondary reagent, and immunoreactions were visualized using a Liquid DAB+ Substrate Chromogen System (cat. no. K3468; Dako). The cell nuclei of sections were counterstained with Mayer’s Hematoxylin (cat. no. 3000-2; Muto Pure Chemicals Co., Ltd.). After dehydration, the sections were mounted in MOUNT-QUICK (Daido Sangyo Co., Ltd.). Microscopic images were captured with an EVOS FL Auto Imaging System (cat. no. AMAFD1000; Life Technologies).

### Assessment of CNVs

Genomic DNA (gDNA) was extracted using a DNeasy Blood & Tissue Kit (Qiagen, Valencia, CA, USA) according to the manufacturer’s protocol. Briefly, 250 ng gDNA was digested with the restriction enzyme *Nsp*I. Digested DNA was ligated into the *Nsp*I adapter and amplified via PCR. The PCR products were purified and fragmented with DNaseI. The fragmented products were end-labeled with biotin and hybridized to Karyostat HD Arrays (Thermo Fisher Scientific) in a GeneChip Hybridization Oven 645 (Thermo Fisher Scientific) overnight. Arrays were washed and stained using a GeneChip Fluidics Station 450 (Thermo Fisher Scientific) and scanned using a GeneChip Scanner 3000 7G (Thermo Fisher Scientific).

Scanned data files were generated using GeneChip Command Console Software and analyzed using Chromosome Analysis Suite v3.2 (ChAS; Thermo Fisher Scientific) considering over 100 kb and at least 50 markers for gains and losses.

### Whole-genome Sequencing

Whole-genome sequencing for all iPSC clones and their derivatives, except H9 ESCs, was performed at CiRA as described previously (49). Briefly, 200 ng gDNA was used as the starting material to generate sequencing libraries, except for in 1210B2 cells. For 1210B2 cells, 420 ng gDNA was prepared as the starting material. WGS libraries were generated using a KAPA Hyper Prep Kit (Kapa Biosystems) without PCR from fragmented gDNA sheared with Covaris LE220 (Covaris). WGS libraries, except for those from 1210B2 cells, were sequenced with HiSeq 2500 (Illumina, San Diego, CA, USA) in 126 paired-end mode. WGS libraries from 1210B2 cells were sequenced with NovaSeq 6000 (Illumina) in 151 paired-end mode. FASTQ files were generated using bcl2fastq v2.17.1.14 and v2.20.0.422 (Illumina) for HiSeq and NovaSeq, respectively. After performing adapter trimming with cutadapt (1.1029 for HiSeq) or fastp (0.20.1 for NovaSeq), FASTQ files were mapped to the reference human genome (hg19). Target coverage was >50 (Table 1A–G). SNVs, insertions, and deletions were called with Genomon (1.0.1) and Genomon2 (2.3.0). Mutations were annotated using ANNOVAR (2016Feb01) and restricted on the coding sequence and splicing regions. To further extract potentially pathogenic mutations, synonymous mutations were excluded, and mutations annotated with COSMIC Cancer Gene Census (ver. 88) and Shibata’s List were assessed. For each analysis batch, called mutations were merged, and variant allele frequency (VAF) values were recalculated. The data containing information that could compromise donor privacy are not publicly available. Some data could be shared upon reasonable request to CiRA-Foundation https://www.cira-foundation.or.jp/e/.

**Table 1:**
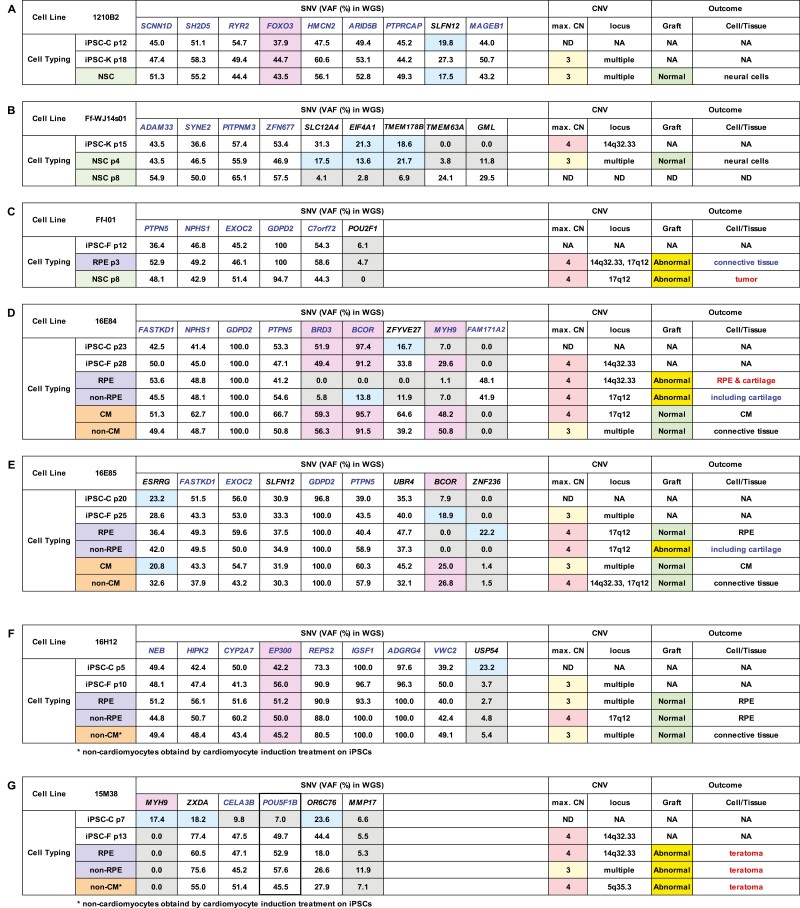

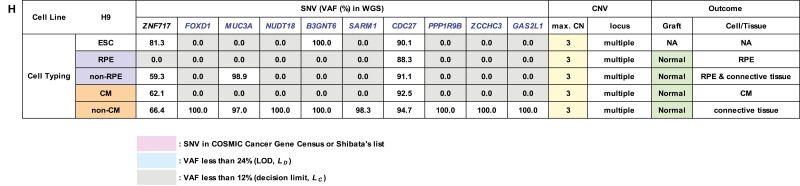
Genetic profiles of iPSC clones and their outcomes after transplantation into NOG mice.

VAF (%) of genes listed in the COSMIC ver.88 database determined by WGS and copy number variants of iPSC clones 1210B2 (A), Ff-WJ14s01 (B), Ff-I01 (C), 16E84 (D), 16E85 (E), 16H12 (F), or 15M38 (G) and their derivatives are shown in the table. Genetic mutations detected by WGS are SNVs, except deletions in *BCOR* in 16E84 (D) and 16E85 (E). VAFs less than 24% (below the detection limit [LOD]) are shown in blue cells, and VAFs less than 12% (below the decision limit) are shown in gray cells. VAFs of genes in the Census database and Shibata’s List showing values above the LOD are highlighted with a pinkish color. Gene whose VAFs reached around 50% or 100% related to the clonality of cells are shown in blue. VAFs of *POU5F1B* in 15M38 suggesting the integration of extrinsic *POU5F1* (thick border). H9 ESCs and their derivatives (H) were used as controls. Maximum copy number (max.CN) of relevant cell clones and the loci showing CN = 4, if detected, are added to the table. Notable or abnormal findings of the transplants are described in blue or red in the table where applicable (Fig. 2). p: passage number, NA: not applicable, ND: not determined, NSC: neural stem cell, iPSC-C: iPSC cultured in CiRA, iPSC-K: iPSC transferred to Keio University used for differentiation and engrafting, iPSC-F: iPSC transferred to Foundation for Biomedical Research and Innovation (FBRI) used for differentiation and engrafting, RPE: retinal pigment epithelial cell, CM: cardiomyocyte. Many of the VAF values for the genes listed in the table reached nearly 50% in autosomal chromosomes and nearly 100% in the X chromosome if a male sample was used, suggesting that all of the iPSC clones tested in this study consisted of clonal expansion from a single cell and its derivatives.

Non-cardiomyocytes obtained by cardiomyocyte induction treatment on iPSCs.

WGS for H9 ESCs was performed by Kurabo Biomedical in accordance with the protocol. Briefly, 1 μg gDNA (TruSeq DNA PCR-free library) was fragmented using a Covaris system. NovaSeq6000 (Illumina) was used for sequencing. The BCL/cBCL (base calls) binary was converted into FASTQ using the Illumina package bcl2fastq2-v2.20. First, paired-end sequences generated using a HiSeq instrument were mapped to the human genome using Isaac Aligner (iSAAC-04.18.11.09) where the reference sequence was the UCSC assembly with reference sequence hg19 and the COSMIC ver 88. Strelka (2.9.10) was performed to identify SNVs and short insertions and deletions. Variants from variant-only VCF files were annotated using SnpEff (v4.3t). Then, in-house programs and SnpEff were applied to annotate the VCF file with additional databases, including ESP6500, ClinVar, and dbNSFP3.5. VAF data generated with QUAL greater than or equal to 1500 (max. 3070) and mutations in exons with a Total_DP(COV) greater than 30 are shown in Table 1H.

### Quantitative PCR

Expression of self-renewal factors was determined by qRT-PCR. Total RNA was extracted using an RNeasy Micro Kit (Qiagen) according to the manufacturer’s instructions. For qRT-PCR, 500 ng DNase-treated RNA was reverse transcribed into cDNA using a QuantiTect Reverse Transcription Kit (Qiagen). qRT-PCR was performed in triplicate using TaqMan Fast Advanced Master Mix (Thermo Fisher Scientific) on a StepOnePlus PCR system (Thermo Fisher Scientific). Relative quantification was calculated using the 2^−ΔΔCt^ method after normalization to glycelaldehyde-3-phosphate dehydrogenase expression.

Genomic integration of vector fragments was determined by genomic PCR. DNA was extracted from cells using a Dneasy Blood & Tissue kit (Qiagen). The primers used in this study are listed in Supplementary Table S1. RT2 SYBR Green qPCR Master Mix (Qiagen), EpiTect ChIP qPCR Primer (Qiagen), and StepOnePlus (Thermo Fisher Scientific) were used for each PCR step.

Copy numbers of the designated loci were evaluated using droplet digital PCR (ddPCR). DNA was extracted from cells with a Dneasy Blood & Tissue kit (Qiagen). Next, 2× ddPCR Supermix (cat. no. 186-3024; Bio-Rad Laboratories, Hercules, CA, USA), 20× Primer & Taqman Probe Mix (Thermo Fisher Scientific), 30 ng DNA, 2 Ut HindIII, 20× Reference Primer & Taqman Probe Mix (Thermo Fisher Scientific; RNase P; cat. no. 440326), primers, and distilled water were mixed to a total volume of 20 mL reaction cocktail. The cocktail was emulsified with Bio-Rad Droplet Generator Oil (cat. no. 186-3005; Bio-Rad Laboratories) in a Bio-Rad QX100 Droplet Generator (cat. no. 186-3001; Bio-Rad Laboratories) according to the manufacturer’s instructions. PCR was performed in a Bio-Rad C1000 thermal cycler (cat. no. 185-1197; Bio-Rad Laboratories) in accordance with the manufacturer’s instructions. Reactants were analyzed using a Bio-Rad QX100 Droplet Reader (cat. no. 186-3001; Bio-Rad Laboratories) and QuantaSoft software (v1.3.2). The copy numbers at designated loci of the samples were calculated after normalization to the signal for the ribonuclease P RNA component H1 (H1RNA) gene (*RPPH1*) on 14q11.2 as a stable control for diploid copies. The primers used are listed in Supplementary Table 1.

### Statistical Analysis

Categorical variables were analyzed with Hayashi’s quantification method type II^27,49^

(https://www.ism.ac.jp/editsec/aism/pdf/003_2_0069.pdf; https://europepmc.org/backend/ptpmcrender.fcgi?accid=PMC1637943&blobtype=pdf) using Excel Statistics ver.7.0 (Esumi Co. Ltd.) to calculate predictivities of the absence or presence of SNVs/del in genes on the Census database and Shibata’s List or CNVs (> 3 copies) for histological abnormalities in differentiated cells derived from iPSCs.

## Results

### Genetic Profiles of iPSC Clones Changed Constantly During Culture

Genetic profile of cells in culture differs in each cell that means genetic profiles of cells in culture are diverse. However, cells showing proliferation potential in the given culture will become dominant in several passages, which may serve to converge the diversity of genetic profiles of cells in the culture as a whole. In this context, the result of whole-genome sequence of cells in culture would change by changing the culture condition, if certain cells show proliferation advantage in the new culture condition. The VAF percentage is determined by the number of sequence reads harboring mutations in genes relative to the total reads, and the VAF (%) profile can be used as a genetic marker of dominant cell population in the given culture, providing information regarding the genetic stability of cells in long term culture by measuring chronological changes in VAF profiles, and the degree of genetic heterogeneity of the cell line by comparing the VAF profiles of cells maintained in different culture systems.^5^ To explore the relationships between genetic abnormalities and tumorigenic events after transplantation, we prepared 5 iPSC test clones with genetic mutations in the Census oncogene database and Shibata’s list (16E84, 16E85, 16H12, 15M38, and 1210B2), which were selected with the expectation that abnormal tissues would be generated after transplantation of the iPSC derivatives, and 2 iPSC clones (Ff-WJs1401, Ff-I01) without mutations in the listed oncogenes. H9 embryonic stem cell derivatives without mutations in the Census oncogene database and Shibata’s list were used as normal transplantation controls. Notably, sequence errors in whole-genome sequencing (WGS) and whole-exome sequencing (WES) may occur, and the validity of VAF values, the limit of detection (LOD), and the decision limit of VAF are directly related to interpretation and judgment of the presence or absence of single-nucleotide variants (SNVs) or deletions. We calculated the LOD and decision limit based on the relative standard deviations (RSDs) of VAFs obtained by WGS and WES for common SNVs/deletions (SNVs/del) in 16E84, 16E85, 16H12, and 15M38 and their derivatives, as previously reported^20,21^ and as shown in Supplementary Fig. S2. Additionally, we set a VAF of 24% as the LOD in WGS, yielding an RSD of 0.3 (ie, 1/3.3) and a VAF of 12% as the decision limit, yielding an RSD of 0.61 (ie, 1/1.65; Supplementary Fig. S2C, D).

The VAF values of the genes in 1210B2 (Table 1A), Ff-WJ14s01 (Table 1B), Ff-I01 (Table 1C), 16E84 (Table 1D), 16E85 (Table 1E), 16H12 (Table 1F), and 15M38 (Table 1G) reached nearly 50% in autosomal chromosomes and nearly 100% in the X chromosome if a male sample was used, and most of these VAF values were maintained throughout the culture period, even after differentiation. These findings suggested that all of the iPSC clones examined in this study originated from single cells and that the majority of cells in culture consisted of clonal expansion from the single cell and its derivatives acquiring additional genetic mutations during culture, owing to their superior growth advantage over other cells under the given culture conditions.

Changes in the VAF profiles of iPSC clones maintained in the undifferentiated state can be used to assess genetic heterogeneity in iPSC clones. The VAF profiles of undifferentiated iPSC clones 16E84, 16E85, and 15M38 having mutations in the Census database varied; for example, the VAF for *MYH9* increased from 7.0% (below the decision limit) to 29.6% (above the LOD) in 16E84-iPSCs (Table 1D), that of *BCOR* increased from 7.9% (below the decision limit) to 18.9% (above the decision limit) in 16E85-iPSCs (Table 1E), and that of *MYH9* dropped from 17.4% (above the decision limit) to 0% in 15M38-iPSCs (Table 1G) during culture. Notably, the mutation in *BCOR* found in clones 16E84 and 16E85 was a deletion (Tables 1D, 1E), and the other mutations in all tested cell lines were SNVs. Change in the VAF profiles of cells cultured with the same medium suggested that cells in culture may undergo constant mutation and that the proportions of cell populations may change if such mutations affected the proliferation potential. In some cases, the VAF profiles changed definitively before and after differentiation. For example, the VAF for *BCOR* dropped from 91.2% to 0%, that for *BRD3* dropped from 49.4% to 0%, and that for *MYH9* dropped from 29.6% to 1.1% when 16E84-iPSCs differentiated into retinal pigment epithelial cells (RPEs; Table 1D). Similarly, the VAF for *BCOR* dropped from 18.9% to 0% when 16E85-iPSCs differentiated into RPEs (Table 1E), suggesting that cells in culture included a variety of cells owing to constant acquisition of mutations, despite being derived from a single cell. Additionally, these findings suggested that specific cell populations benefitting from the RPE differentiation protocol expanded and became dominant during the long culture period (3 months).

### Relationship Between Mutations in Genes from the Oncogene Lists and Histological Outcomes after Transplantation

Next, we explored whether mutations in the Census oncogene database and Shibata’s List were related to abnormal tissue or tumor formation after transplantation. For this, we prepare 2 types of animal transplantation models. One was a subcutaneous transplantation model using severely immunodeficient NOG mice, which showed no T, B, or natural killer cell activity and had dysfunctional dendritic cells and macrophages^19^; this model enabled us to monitor the engraftment of 1 × 10^6^ cells embedded in Matrigel for 6-12 months.^22^ This model is not a tumorigenicity test that is used in the clinical setting, but serves as a long-term QC test for in vivo culture when using mice as the ultimate incubator. Such results cannot be obtained using in vitro culture assays; thus, the model has the advantage of permitting the histological examination of a large numbers of transplants.

In a previous study, tumor detection sensitivity of subcutaneous and clinical (subretinal) routes was compared by calculation of the minimum tumor-producing dose among half of the animals transplanted (TPD50). In our previous study of iPSC-derived RPEs (iPSC-RPEs), the TPD50 value of HeLa cells, which were used as a positive, in the subcutaneous NOG mouse model was 12.6 cells, which was comparable to that in the subretinal nude rat model (21 cells). By contrast, the TPD50 values in a subcutaneous mouse model of iPSC transplantation (201B7) and a nude rat subretinal engraft model were 132 and 5.0 × 10^4^ cells, respectively^23^; these differences were attributable to the secretion of pigment epithelium-derived factor from RPEs, resulting in induction of apoptosis in iPSCs.^24^

**Figure 1.**
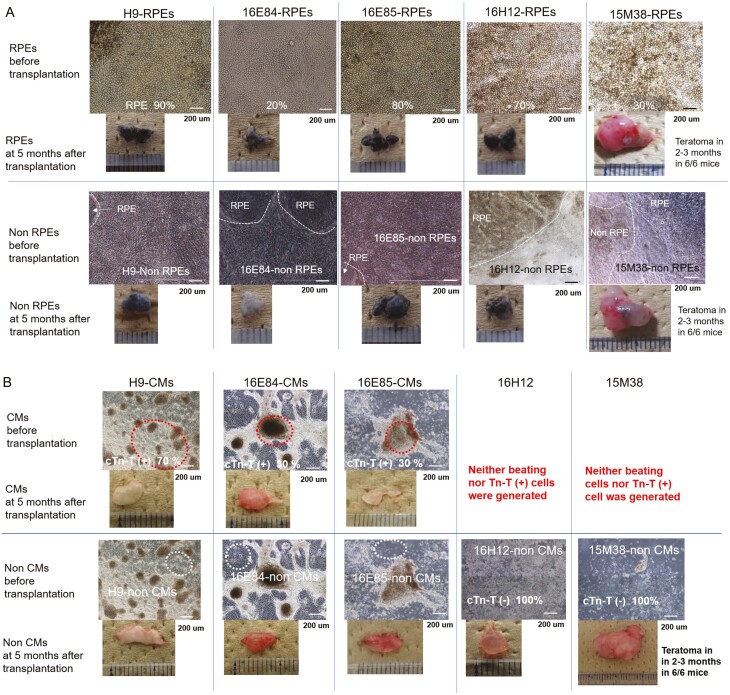
Morphology of RPEs or cardiomyocytes derived from iPSC clones and their transplants before transplantation. (A) iPSCs 16E84, 16E85, 16H12, and 15M38 or H9 ESCs (control) were cultured using an RPE differentiation protocol. Cells differentiated into RPEs were termed iPSC-RPEs or H9-RPEs (upper panels) and cells other than RPEs were termed iPSC-non RPEs or H9-non RPEs (lower panels). RPEs or non RPEs derived from iPSCs prior to transplantation and relevant transplantation after 5 months of monitoring are shown in upper and lower panels, respectively. The differentiation efficiency to RPEs in the whole culture dish is indicated as a percentage and appended in the photos of RPEs. (B) iPSCs 16E84, 16E85, 16H12, and 15M38 or H9 ESCs (control) were cultured with a cardiomyocyte (CM) protocol. Cells differentiated into CMs (red dotted line) were termed iPSC-CMs or H9-CMs (upper panels) and cells other than CMs (White dotted line) were termed iPSC-non CMs or H9-non CMs (lower panels). CMs or non CMs derived from iPSCs prior to transplantation and relevant transplantation after 5 months of monitoring are shown in upper and lower panels, respectively. The differentiation efficiency to CMs in whole culture dishes was assessed by immunostaining with cardiac troponin T and is shown as a percentage appended in the photographs in the upper panels.

**Figure 2.**
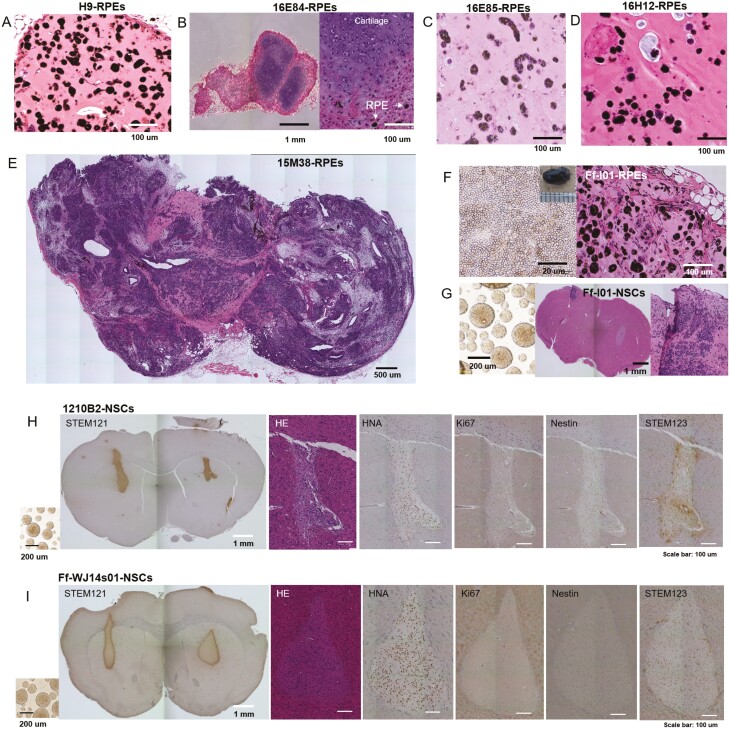
Histology of transplants of iPSC-derived retinal pigment epithelial cells (RPEs), cardiomyocytes (CMs), and neural stem cells (NSCs). Histological analysis of transplants. HE staining of (**A**) H9-RPE-, (**B**) 16E84-RPE-, (**C**) 16E85-RPE-, (**D**) 16H12-RPE-, or (**E**)15M38-RPE-transplants is shown. (**F**) Ff-I01-RPEs before transplantation (left) and section of the transplant. (**G**) Ff-I01-NSCs before transplantation (left), and mouse brain section stained by HE. (**H**) 1210B2-NSCs or (I) Ff-WJ14s10-NSCs before transplantation are shown on the left. Mouse brain sections 6 months after transplantation were stained with STEM 121, HE, anti-HNA antibodies, Ki67, anti-Nestin antibodies, and STEM123 (from left to right).

In the current study, considering the number of animals to be used for transplantation (6-8 mice per transplantation group) and the sensitivity required to detect the formation of abnormal tissues, we used a subcutaneous NOG mouse transplantation model to examine the formation of abnormal tissues and the histology of transplants of iPSC-RPEs (tissue differentiated into RPEs), iPSC-non RPEs (tissue not differentiated into RPEs; Fig. 1A), iPSC-derived cardiomyocytes (tissue differentiated into CMs), and iPSC-non CMs (tissue not differentiated into CMs; Fig. 1B) derived from several clones (Tables 1C–G). The morphology, origin, and doubling time of 16E84-iPSCs, 16E85-iPSCs, 16H12-iPSCs, and 15M38-iPSCs before differentiation are shown in Supplementary Fig. S1A. Notably, 16H12-iPSCs and 15M38-iPSCs could differentiate into RPEs, but not into CMs (Fig. 1), suggesting a tendency to differentiate into a specific lineage and demonstrating that a cardiomyocyte differentiation protocol could be used to eliminate cells with differentiation bias. H9-RPEs with no mutations in the Census database and Shibata’s List showed normal RPE morphology (Fig. 2A); these cells were used as positive controls of RPE transplantation derived from the other iPSC lines.

Based on the decision limit (12%, Supplementary Fig. S2) in WGS, we judged that 16E84-RPEs without detectable SNVs/del in cancer-related genes generated cartilage tissue in the transplant (Fig. 2B). 16E85-RPEs with no mutations in the Census database did not show abnormal RPE tissue after transplantation (Fig. 2C). 16H12-iPSCs having mutations in acetyltransferase EP300 differentiated into normal RPEs (Fig. 2D). By contrast, 15M38-RPEs, 15M38-non RPEs, and 15M38-non CMs, which had no mutations in relevant oncogenes from the Census database or Shibata’s List, but carried mutations in *POU5F1B* before transplantation (Table 1G), formed teratomas in mice (Fig. 2E, S1B, S1C). Because the sequence of mutated *POU5F1B* was similar to extrinsic *POU5F1*, which was used for reprogramming, unexpected integration of the episomal vector was examined, and the EBNA sequence in the plasmid was detected (Supplementary Fig. S1D). One possible cause of this observed teratoma formation was persistent expression of extrinsic *POU5F1* integrated into host DNA (Supplementary Fig. S1D). However, more evidence is needed to conclude that accidental integration of extrinsic *POU5F1* was the sole reason for teratoma formation, and we cannot deny the possibility that at least one mutation in *POU5F1B* in 15M38 derivatives and genetic instability (carrying the maximum copy number of more than 3 at 14q32.33 or 17q12) could cooperatively contribute to teratoma formation. The integration of extrinsic *POU5F1* was not intentional, and the aim of this in vivo cell transplantation experiment was to examine the detection of mutations in cancer-related genes and the prediction of abnormal tissue formation after transplantation; thus, we included cases of 15M38 derivatives without detectable SNVs for further analytical studies. Ff-I01-RPEs had no mutations from the Census database before transplantation (Table 1C); however, RPE transplantation resulted in abnormal tissue formation, including non-RPE cells (Fig. 2F, S1E).

The second model was the trans-striatum implantation model for iPSC-derived neural stem/progenitor cells (hereinafter called neural stem cells [NSCs]).^18,25^ Because subcutaneously engrafted NSCs cannot survive, even when embedded with Matrigel, in the transplant for more than 1 week, a trans-striatum transplantation model that provides a microenvironment similar to that in the spinal cord was applied based on the previous animal studies of neural diseases.^26^ In this experiment, Ff-I01-NSCs showed no mutations in the Census database or Shibata’s List before transplantation (Table 1C), but generated tumors (Fig. 2G). WGS of ex vivo culture of transplants at passage 3 (p3) and p8 (Supplementary Fig. S1E) and in vitro culture at p10 (Supplementary Fig. S1F) did not result in major alterations in genetic profiles; however, propagation of karyotype abnormalities was observed in Ff-I01-NSCs after prolonged in vitro culture (p10). 1210B2-NSCs had mutations in the *FOXO3* gene listed in the Census database (Table 1A), but the engrafted transplant showed no abnormalities, with mature glia cells and few proliferative or Nestin(+) immature cells  (Fig. 2H). Ff-WJ14s-NSCs showed no mutations in the Census database or Shibata’s List (Table 1B) and showed normal engrafted tissue, similar to engraftment of 1210B2-NSCs (Fig. 2I).

In a series of transplantation experiments, we did not find correlations between mutations in the Census database or Shibata’s List with the formation of abnormal tissues or tumors in NOG mice. Then, predictivity of the detection of SNVs/del in cancer-related genes for abnormal tissue formation, including tumorigenesis, was analyzed among cell clones differentiated into target cells (RPEs, CMs, or NSCs). Thus, these findings may be useful for “go/no-go” clinical decision making by Institutional Review Boards. Hayashi’s quantification method type II^27^ was used for statistical analyses (Table 2).  In final target cells (RPEs, CMs, or NSCs) for transplantation, no deletions were detected; however, some cells had cancer-related SNVs/del. Notably, abnormal tissues were generated more frequently from iPSC-derived final products without mutations than from those with mutations. Among 5 abnormal transplants, including tumors, no tissues were generated from iPSC-derived products with cancer-related SNVs/del, and all 5 cases of abnormal tissue formation were generated from iPSC-derived products with no detectable cancer-related SNVs/del. By contrast, among 9 normal transplants, 5 tissues were generated from iPSC-derived products with cancer-related SNVs/del and 4 tissues were generated from iPSC-derived products with no detectable cancer-related SNVs/del. The test specificity and sensitivity were 44% and 0%, respectively. The overall predictability of the occurrence of abnormal tissue after transplantation based on the detection of SNVs/del in iPSC derivatives was 29% (<50%), with a correlation ratio of *η* = 0.56.

**Table 2.**
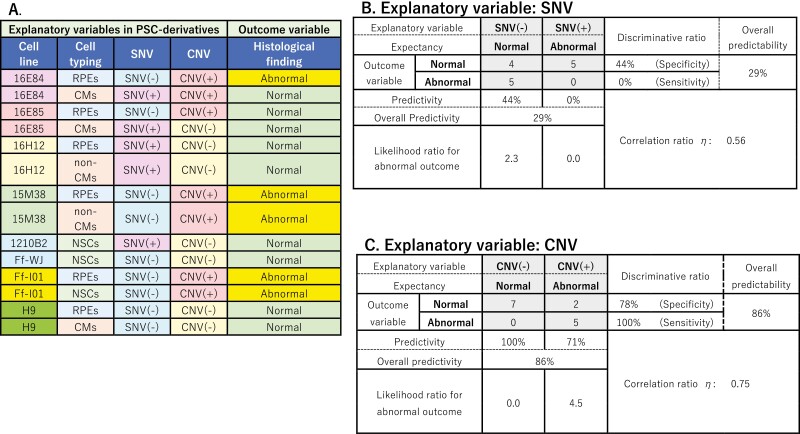
Outcome of the transplants of the cells with SNVs in the cancer-related genes or with copy number exceeding 3.

A. Table of iPSC-derivatives and their histology after transplantation. Cell typing: identities of differentiated cells transplanted, RPEs: retinal pigment epithelial cells, CMs: cardiomyocytes, NSCs: neural stem cells. All of the mutations in cancer-related genes listed in the table are SNVs, except deletions in *BCORE* in 84-CMs or16E85-CMs. SNV (+): cells with SNVs/del in cancer-related genes, SNV (−): cells with no detectable SNVs/del in cancer-related genes, CNV (+): cells with a maximum copy number exceeding 3, CNV (−): cells with copy numbers of 3 or below. B: Predictivity of abnormal tissue formation after transplantation by detecting cancer-related SNVs/del in the final product. C: Predictivity of abnormal tissue formation after transplantation by detecting a maximum copy number exceeding 3 in the final product. Categorical variables were analyzed with Hayashi’s quantification method type II.

### Abnormal Histological Images Could Be Partly Evaluated by Tracing Changes in Copy Number Variants (CNVs)

We presumed that not all cancer-related mutations may cause abnormal tissue or tumor formation after transplantation, and our series of in vivo transplantation studies supported this idea. Therefore, we next performed additional genetic tests to predict the formation of abnormal tissues after transplantation and evaluated whether the CNV profile could be used as such a QC test. Accordingly, we used an SNP array (Karyostat HD; Thermo Fisher Scientific, MA) that could detect copy numbers at loci of 50 kb or more with 2.5 million probes to quantitatively evaluate CNVs. Notably, the sites of CNVs changed during the differentiation process (Supplementary Figs. S3, S4); that is, some sites became hard to detect, whereas others were newly detected, suggesting the profiles of CNVs could serve as a genomic marker of the dominant cell population in the given culture. Additionally, this dominant population may change if the acquired de novo mutations affected the proliferation potential of the cell during culture in the undifferentiated state or if the new culture medium for differentiation selectively supported the growth of a specific cell population. Although cells in culture had CNVs at various loci, their copy numbers seldom exceeded 3 (Supplementary Figs. S3, S4). Moreover, 2 common hotspot loci with a copy number of 4 were detected in iPSC clones and their derivatives, regardless of culture medium or differentiation status. These loci were 14q32.33 (16E84-CMs, 16E84-non-RPEs, 16E85-non-RPEs, 15M38-iPSCs, and all derivatives, ie, Ff-WJ14s01, Ff-I01-RPEs, and Ff-I01-NSCs) and 17q12 (16E84-iPSCs, 16E85-non-CMs, 16E85-RPEs, 16E85-non-RPEs, 16H12-non-RPEs, and Ff-I01-RPEs; Table 1 and Supplementary Figs. S3, S4). Digital droplet DNA polymerase chain reaction (PCR) for the amplicons covering the locus at Chr.14:106260714 in Chr.14q32.33 or Chr.17:36120285 in Chr.17q12 (GRCh38) was performed (Supplementary Fig. S5), and the results supported the findings of the CNV array. The predictivity of CNVs for abnormal tissue formation was analyzed among cell clones differentiated into the target cells (RPEs, CMs, and NSCs), and the results are shown in Table 2C. Out of 5 abnormal transplants, 5 tissues were generated from iPSC-derived products with a copy number of 4 (discriminative ratio [=test sensitivity] was 100%). Additionally, out of nine normal transplants, 7 tissues were generated from iPSC-derived products with a copy number of 3 or less. The test specificity and sensitivity were 78% and 100%, respectively. The overall predictability of abnormal tissue formation by detection of a copy number greater than 3 was 86% with as correlation ratio *η* of 0.75.

## Discussion and Conclusion

WGS analyses by next-generation sequencing (NGS) have provided definitive information regarding genomic mutations and variations related to cell and tissue function, the diagnosis of congenital disease, and the prognosis of patients with cancer. However, interpretation of the phenotype of transplanted tissue may be confusing if prediction of the outcome of transplanted cells is performed by WGS alone, without epigenetic, gene expression, or microenvironmental information. One reason may be that an oncogene exerts its functions in the context of the microenvironment or developmental stage of the tumor, and if the new environment surrounding the cells after transplantation is different from the original microenvironment of the cells, the behavior of the oncogene and the outcomes of cell transplantation may be unpredictable. Notably, the results of WGS may also vary depending on the sequencing method, reagents, analysis software, and coverage depth as well as the version of the reference genome assembly (GRCh37 or GRCh38). The LOD or decision limit of VAFs scored needs to be discussed to make an appropriate interpretation of WGS/WES results. Indeed, several reports have shown that WGS/WES sensitivity for detection of SNVs can vary by the sequence error rate, coverage rate, and detected variant reads.^21,28,29^ The standard read depth of 30-50× with WGS has been reported to be insufficient to detect SNVs with rates of <15% for analysis of tumor complexity.^30^ Moreover, one report showed that calculation of the LOD of VAF should be performed with recommended coverage depth and minimum of variant reads to minimize the probability of false-positive and false-negative results.^31^ Based on their calculations (http://app.olgen.cz/clc/), an LOD of VAF of 24% could be obtained with a recommended coverage of 42 and a minimum variant reads number of 3, consist with our proposed LOD of VAF of 24%, calculated with an RSD of 0.3, as determined by VAF values from approximately 60 reads average. Therefore, judgment of the detection of SNVs/del may vary dramatically based on these sequencing parameters. Furthermore, the cancer-related oncogene Census database is revised frequently as new reports are published. These findings supported the necessity of understanding the limitation of sequence results and the sensitivity of detection to genomic changes (variant types and their allele frequencies) when we interpret genomic/epigenomic data obtained from state-of-the-art technologies, such as NGS. Mutations in the Cosmic Census and Shibata’s List have been retrospectively analyzed and suggested to play some roles in cancer development. Therefore, if the presence of SNVs/del in these lists was positively correlated with abnormal tissue formation in in vivo tumorigenicity tests, the results of WGS testing could reduce the number of animals used in in vivo tumorigenicity tests. In this study, however, no such correlations were observed. This discrepancy suggests that even if each mutation contributes to the health risk, eg, malignant transformation or abnormal tissue formation, the probability of such risks is very low, if not zero. Therefore, when WGS is performed prior to clinical administration, the test results cannot be quantitative risk predictors, but rather should be used as reference information for safety reassurance, as described in the MHLW guideline document.^8^

In this study, we found a positive correlation between the presence of copy number greater than 3 and the formation of abnormal tissues after transplantation; the overall predictability was 86%. CNVs have been used to diagnose genetic abnormalities in congenital diseases and characterization of cancers.^32-41^ Although commercially available SNP-based CNVs arrays are not designed for the detection of genetic structural abnormalities or aberrations generated during long culture periods, several hotspot loci for CNVs have been reported previously.^42,43^ In the current study, all cells in the culture showed genomic loci with CNVs. Although their copy numbers seldom exceeded 3, we found 2 hotspots with copy numbers exceeding 3 at 14q32.33 (*n* = 6), 17q12 (*n* = 6), or both (*n* = 1) among the 24 cases examined, consistent with previous reports showing a copy number of 4 at 14q32.33.^34,36,44^ In particular, 4 of 18 iPSC clones in the Korea National Stem Cell Bank showed a copy number of 4 at either 14q32.33 or 17q12, and no other locus was found to have a copy number greater than 3.^44^ The mechanism for the selective DNA amplification at 14q32.33 and/or 17q12 and the biological impact of this mutation on abnormal tissue formation from iPSC derivatives are currently unclear. One plausible explanation is that the 14q32.33 locus, located at the tip of chromatin, and genes nearby the telomere have a higher chance of amplification to maintain the chromatin structure. Moreover, amplification of *JAG2* encoded in the 14q32.33 locus could confer tumorigenic proliferation to the cells, as has been reported in cancer cells.^45^ The *HER2* gene is encoded in the 17q12 locus, and amplification of *HER2* triggers multiple proliferation signals and drives genomic instability along chromosome 17q, leading to different patterns of gene amplification.^46^ Therefore, amplification of the 17q12 locus confers cells with uncontrolled proliferation, and if cells have 4 copies of *HER2*, the cells would become dominant in the culture.

With regard to QC of iPSC-derived products for cell therapy applications, chronological changes in CNVs may be useful for assessing the genetic instability of cells in long-term culture, and quantitative analysis of copy numbers by CNV arrays can help predict abnormal tissue formation during the first several months after transplantation into immunocompromised animals. Of course, having a copy number of 3 or below is not a sufficient guarantee for the safety of iPSC-derived differentiated cells. In vitro and in vivo tests to detect transformed cells and residual undifferentiated iPSCs, another major hazard for tumorigenicity, should also be performed based on the characteristics and limitations of the assays.^47^ Using these tests, in vitro CNV screening prior to in vivo tumorigenicity testing in immunocompromised animals could significantly reduce the cost and time of development and QC of iPSC-derived products, help to efficiently obtain optimal starting cells, and facilitate manufacturing.

## Supplementary Material

szac014_suppl_Supplementary_Figures

## Data Availability

The data that support the findings of this study are openly available at figshare.com: Figure 1A: 10.6084/m9.figshare.19227939 Figure 1B: 10.6084/m9.figshare.19227864 Figure 2: 10.6084/m9.figshare.19227957 Table 1 and 2: 10.6084/m9.figshare.19228053 Graphical Abstract: 10.6084/m9.figshare.19228023 Supplementary Figures: 10.6084/m9.figshare.19228044
