# Supplementary material for: Correlation Between Genetic Abnormalities in Induced Pluripotent Stem Cell-Derivatives and Abnormal Tissue Formation in Tumorigenicity Tests
Source: Stem Cells Transl Med. 2022 Apr 21;11(5):527–38. doi: 10.1093/stcltm/szac014 (PMC9154342; doi:10.1093/stcltm/szac014)
Supplement: szac014_suppl_Supplementary_Figures [file szac014_suppl_supplementary_figures.pdf]

Fig. S1

A iPSC clones with genomic mutations in the Census database and Shibata's list.

	ESC H9	16E84	16E85	16H12	15M38
ESC/iPSCs maintained in the undifferentiated state with Stemfit AK02N on iMatrix -coated dishes	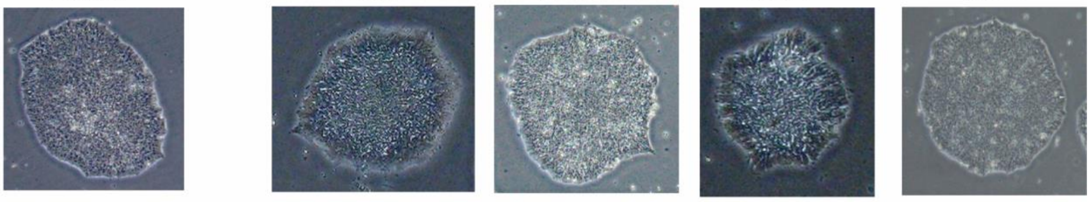				
Passage number at FBRI	P39	P28	P25	P10	P13
Original iPSC clones Origin of cell		QHJI01s01 PBMCs	QHJI01s01 PBMCs	DRXT22 PBMCs	YZWJ24 Cord Blood
Doubling time (n = 4)	23.0 ± 1.5 h	27.8 ± 3.8 h	27.9 ± 4.0 h	21.7 ± 1.2 h	31.0 ± 4.3 h

B HE staining of transplant of H9-non RPEs, 16E84-non RPEs, 16H12-non RPEs and 15M38-non RPEs.

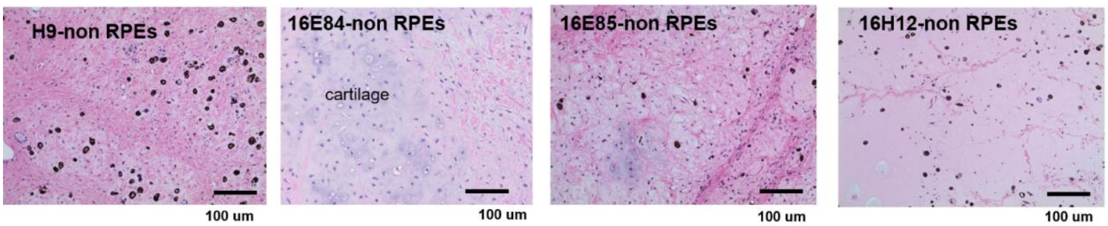

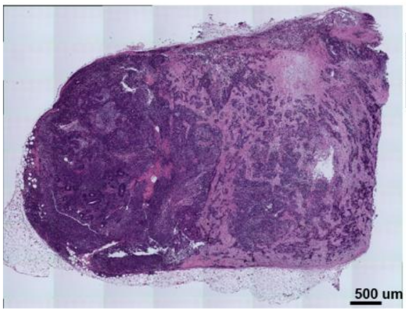

15M38-non RPEs

	ZXDA	*CELA3B*	*POU5F1B*	*OR6C76*	*CREB1*	*MMP17*	*GAREM1*	*ZCCHC6*	max. CN	CN site
transplanted RPEs	8.5%	36.1%	57.4%	29.5%	47.8%	29.2%	0%	0%	4	14q32.33
transplanted non RPEs	11.4%	54.4%	60.0%	34.6%	0%	41.7%	33.3%	0%	3	multiple

C HE staining of transplants of H9, 16E84, 16E85, 16H12, 15M38 in cardiomyocyte lineage

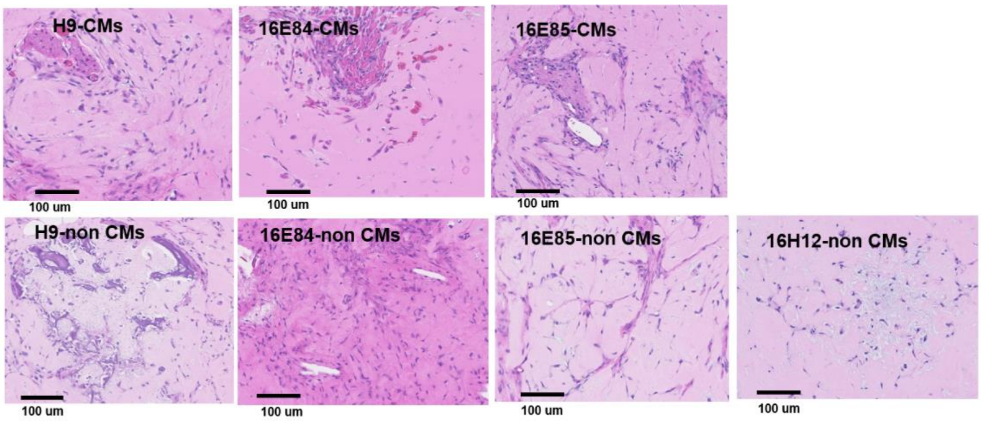

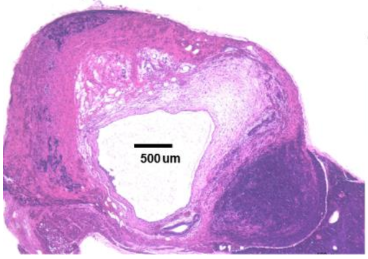

15M38-non CMs

	ZXDA	*CELA3B*	*POU5F1B*	*OR6C76*	*CREB1*	*MMP17*	*GAREM1*	*ZCCHC6*	max. CN	CN=4 site
tansplanted non-CMs	55.3%	52.0%	72.1%	25.6%	0%	63.6%	0%	28.9%	4	14q32.33

D Genomic- and qRT-PCR detection of plasmid and POU5F1, POU5F1B and NANOG expression.

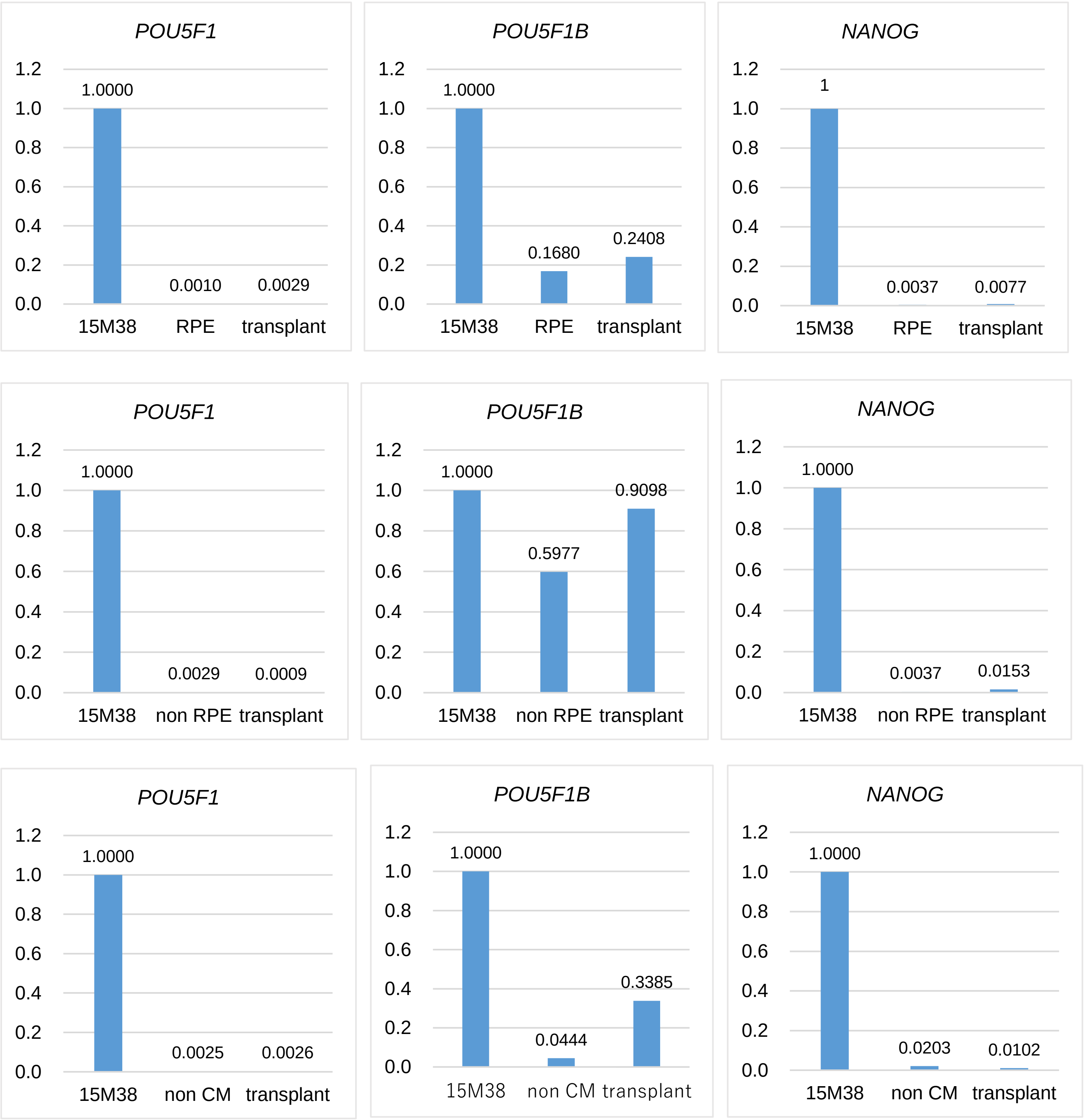

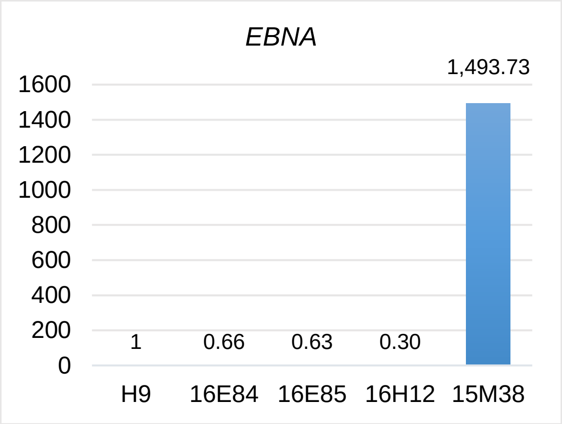

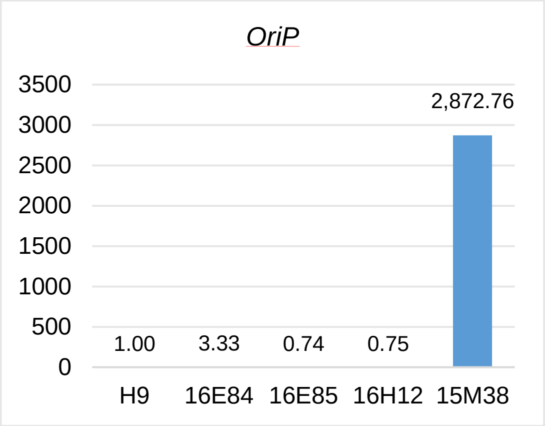

Genome PCR Primer

Gene	F/ R	seq (5'-3')
Orip	F	TTC CAC GAG GGT AGT GAA CC
Orip	R	TCG GGG GTG TTA GAG ACA AC

Gene	Assay ID
GAPDH	GPH1002905(-)

qPCR Primers

Gene	F/ R	seq (5'-3')
EBNA	F	TAT GAC AAA GCC CGC TCC TAC
EBNA	R	TCA CCC TCA TCT CCA TCA CC
GAPDH	F	CCA CTC CTC CAC CTT TGA CG
GAPDH	R	ATG AGG TCC ACC ACC CTG TT

Taq man PCR Primers

Gene	Taqman Probe
POU5F1	Hs04260367_gH
POU5F1B	Hs04995079_g1
NANOG	Hs02387400_g1
GAPDH	Hs02786624_g1

E HE staining of transplants of Ff-I01-RPE or Ff-I01-NSC and VAFs of relevant transplants by WGS.

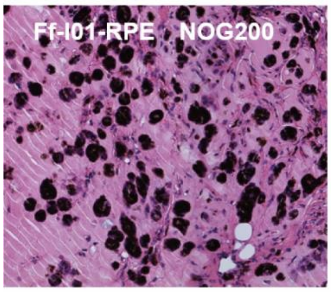

NOG 200									
SVIL	*PTPN5*	*NPHS1*	*EXOC2*	*RANBP9*	*GDPD2*	*C7orf72*	*POU2F1*	*CCNT2*	Max.CN
38.8%	64.7%	62.2%	48.5%	3.0%	100.0%	41.7%	21.3%	3.8%	3

NOG 200 is ID for engrafted mouse.

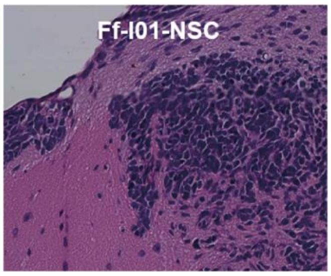

Primary culture of grafts from Ff-I01-NSC with DMEM_F12 p3						
PTPN5	*NPHS1*	*EXOC2*	*GDPD2*	*C7orf72*	*POU2F1*	*GRIN2A*
42.1%	48.0%	50.0%	95.5%	40.0%	1.5%	4.7%

Primary culture of grafts from Ff-I01-NSC with DMEM_F12 p8						
PTPN5	*NPHS1*	*EXOC2*	*GDPD2*	*C7orf72*	*POU2F1*	*GRIN2A*
47.1%	65.2%	48.6%	90.9%	45.7%	0.0%	0.0%

F Transition of genetic instability of Ff-I01-RPE or Ff-I01-NSC and VAFs of relevant transplants by WGS.

Test	Item	Ff-I01 NSCs			
		P3 end	P5 end	P10	After transplantation
Karyotype	Chromosome	46[50]	44[1]/46[48]/47[1]	45[3]/46[37]/47[8]/ 49[1]/55[1]	46[9]/48[11]
	G-band	46,XY[22] (SCA[3])	46,XY[28] (SCA[1])	46,XY[16] 47,XY,+11[7]	46,XY[5]/ 48, XY,+2,+8[3]

**Ff-I01-NSCs in culture p10 (without transplantation)**						
PTPN5	***NPHS1***	***EXOC2***	***GDPD2***	***C7orf72***	***GRIN2A***	***POU5F1B***
**42.9%**	**41.8%**	**37.7%**	**79.3%**	**37.7%**	**3.8%**	**28.6%**

Fig. S1. A: Morphology of iPSC clones with genomic mutations in the Census database and Shibata’s list. B: HE staining of transplanted H9-non RPEs, 16E84-non RPEs, 16H12-non RPEs, and 15M38-non RPEs. Genes listed in the Census database and Shibata's List from hg19/ver88 with VAF (%) values above the detection limit (24%) are highlighted in pink, and those below the detection limit are indicated in gray. VAFs of autosomal dominant genes reaching approximately 50% are shown in blue, indicating the clonality of the cells in the transplant. C: HE staining of transplants of H9, 16E84, 16E85, 16H12, and 15M38 in the cardiomyocyte lineage. Genes listed in the Census database and Shibata's List from hg19/ver88 are highlighted in pink. VAFs below the detection limit are highlighted in gray. VAFs of autosomal genes reaching approximately 50% are shown in blue, indicating the clonality of the cells in the transplant. D: Genomic-PCR and qRT-PCR detection of plasmid expression and *POU5F1*, *POU5F1B*, and *NANOG* expression. E: HE staining of transplants of Ff-I01-RPEs or Ff-I01-NSCs and VAFs of relevant transplants, as determined by WGS. Genes listed in the Census database and Shibata's List from hg19/ver88 are highlighted in pink. VAFs below the detection limit are highlighted in gray. VAFs of autosomal genes reaching around 50% or those of sex chromosomal genes reaching 100% are displayed in blue to show the clonality of cells in the transplant. F: Transition of the genetic instability of Ff-I01-RPEs or Ff-I01-NSCs and VAFs of relevant transplants by WGS. Karyotyping of Ff-I01-NSCs at p10 in culture (without transplantation). Genes listed in the Census database and Shibata's List from hg19/ver88 are highlighted in pink. VAFs below the limit of detection (24%) are shown in gray. VAFs of autosomal genes reaching approximately 50% are displayed in blue, indicating the clonality of the cells in the transplant.

Fig. S2

The detection limit (LOD) and decision limit for VAF determined by WGS and WES

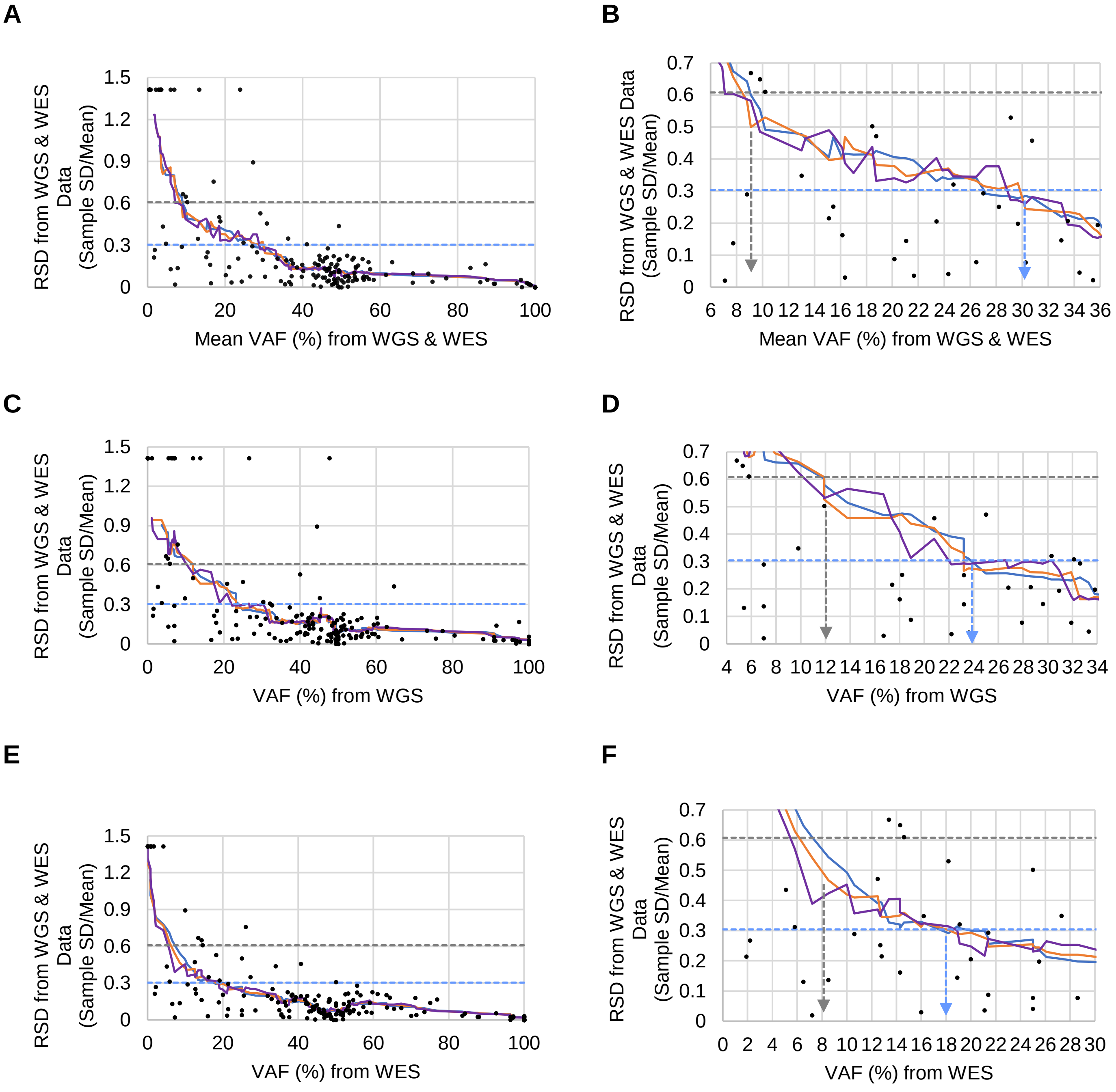

Fig. S2. The detection limit (LOD) and decision limit for VAFs, as determined by WGS and WES. WGS and WES were conducted in parallel. To ensure consistency between the results of the WGS and WES, the LOD (L_D) and decision limit (critical value, L_C) (IUPAC Commission on Analytical Nomenclature, Pure & Appl Chem. 1995;67:1699-1723) for VAFs were examined, based on the relative standard deviations (RSDs) of VAFs obtained by WGS and WES for common SNVs/del in common samples of iPSCs and iPSC derivatives from cell lines 16E84, 16E85, 16H12, and 15M38, according to the method reported by Miura *et al*. (Miura T *et al.,* BMC Genom Data. 2021;22:8). A and B show the relationships between the mean VAFs of SNVs/del measured by WGS and WES and their RSDs. WES was conducted in parallel with WGS, as shown in Table 1. The purple, orange, and dark blue lines represent moving averages of 13, 17, and 21 data points, respectively. In C and D, the RSD of the mean VAF of an SNVs/del measured by WGS and WES was plotted against the VAF of the SNVs/del measured by WGS. In E and F, the RSD of the average VAF of an SNVs/del measured by WGS and WES was plotted against the VAF of the SNVs/del measured by WES. Based on these results, when the average VAF of an SNVs/del measured by WGS and WES was less than or equal to 30% (i.e., its LOD), which gave an RSD of 0.30, the SNVs/del were detected with sufficient certainty, and if the average VAF was greater than or equal to 9% (i.e., its decision limit), which gave an RSD of 0.61, the SNVs/del were detected above the LOD (A and B). When the VAF of an SNV/del was greater than its LOD and decision limit, the VAF of the SNV/del measured by WGS was greater than 24% and 12% in most cases, respectively (C and D). In the current study, these values are referred to as the LOD and decision limit for VAF in WGS, respectively.

Fig. S3

A Profile of CNVs from 16E84-iPSCs and their derivatives

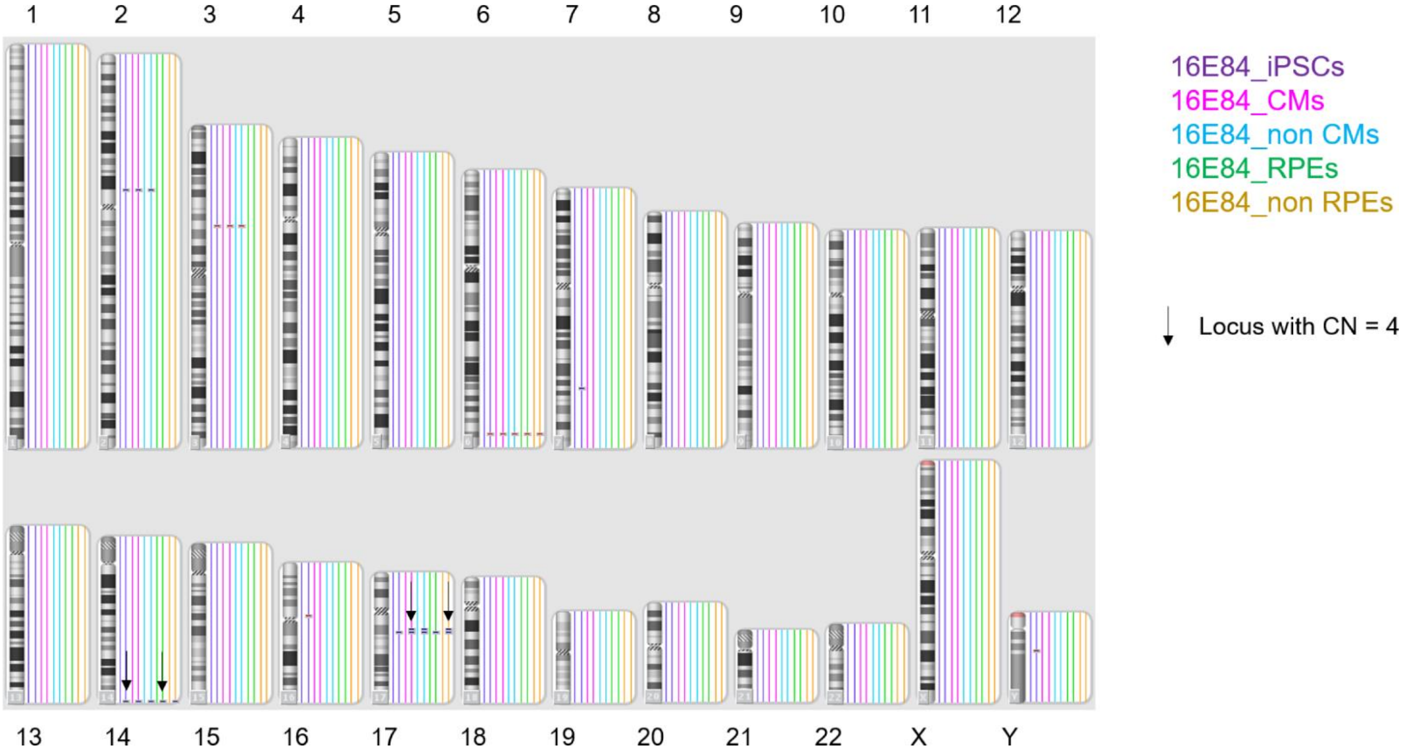

B Profile of CNVs of 16E85-iPSCs and their derivatives

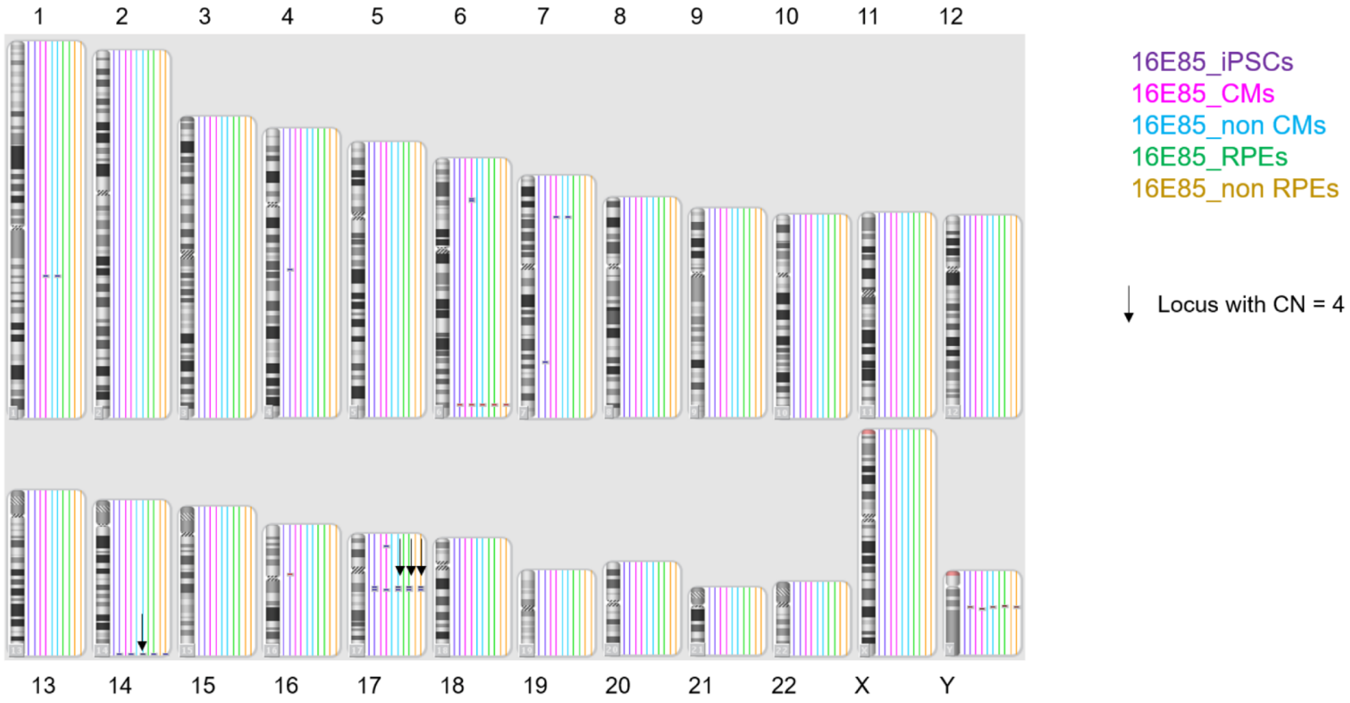

C Profile of CNVs of 16H12-iPSCs and their derivatives

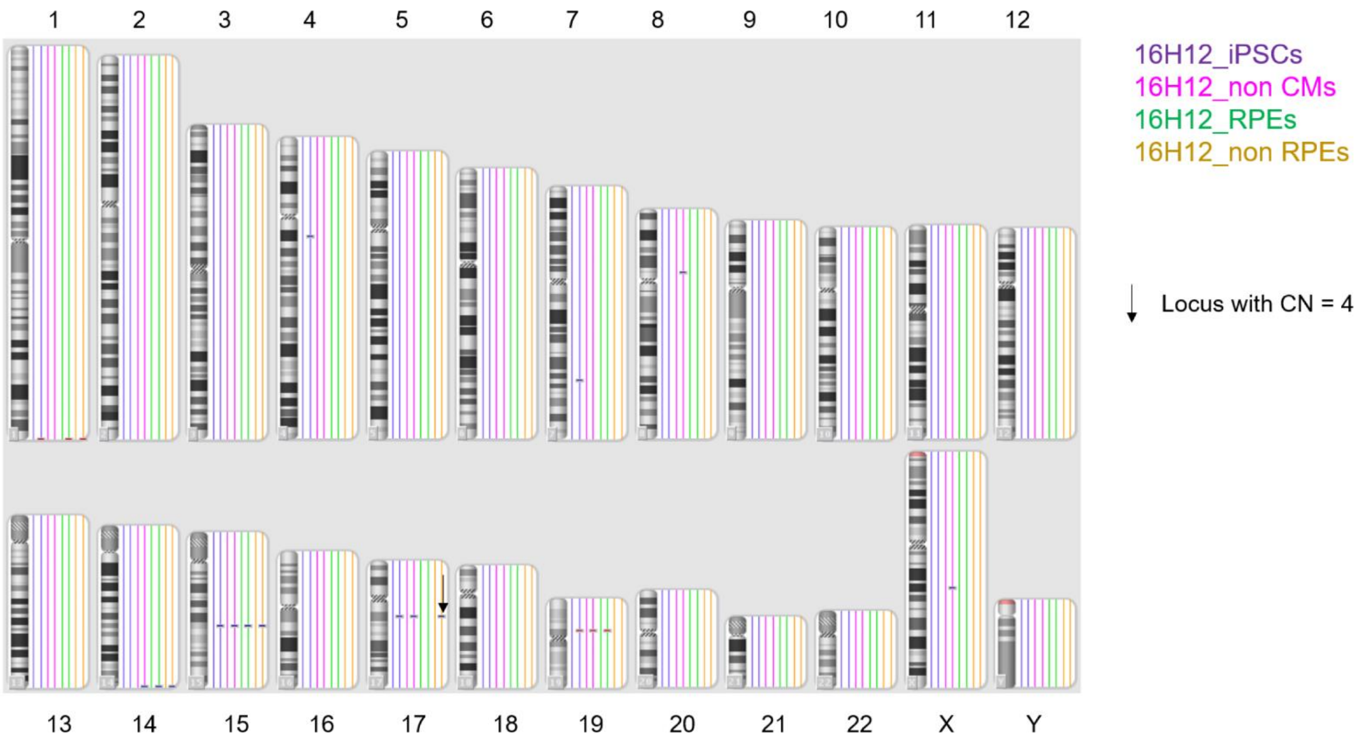

D Profile of CNVs of 15M38-iPSCs and their derivatives

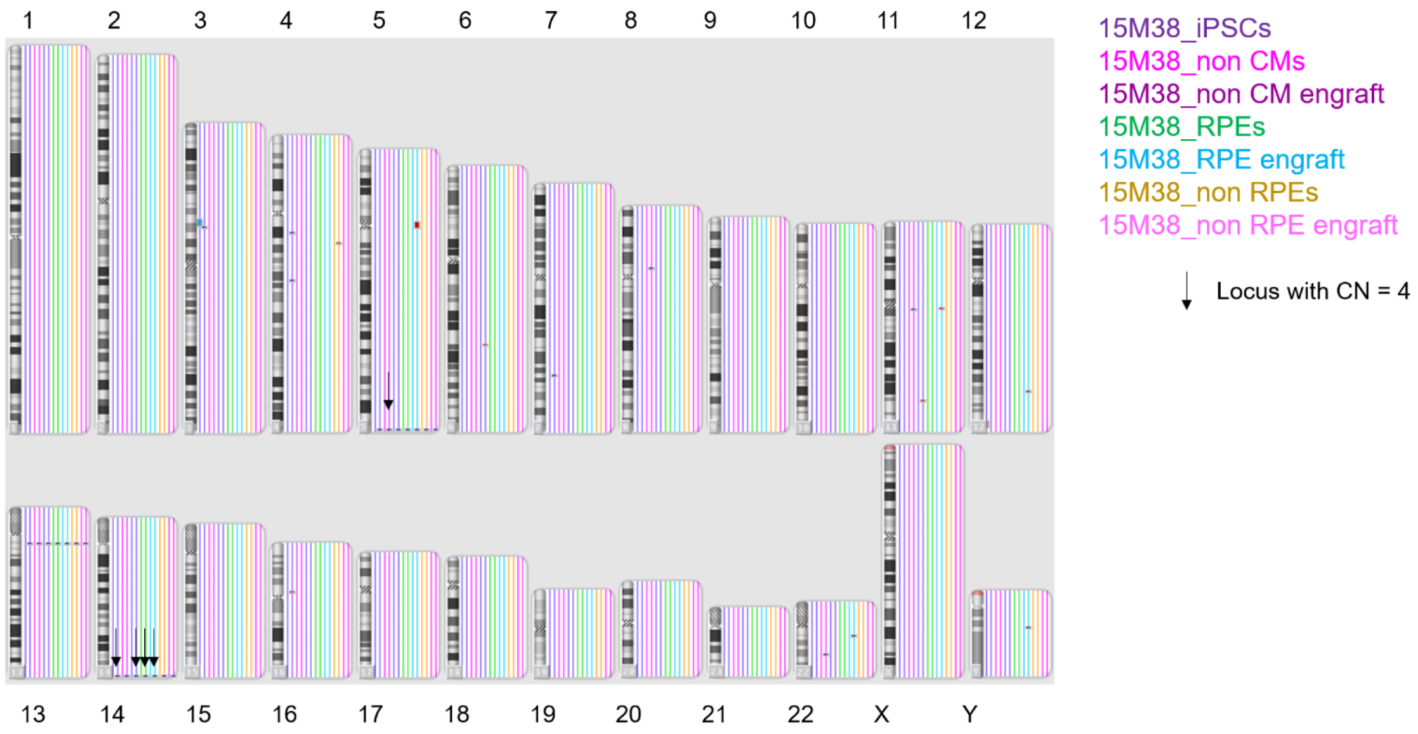

E Profile of CNVs of H9-ESCs and their derivatives

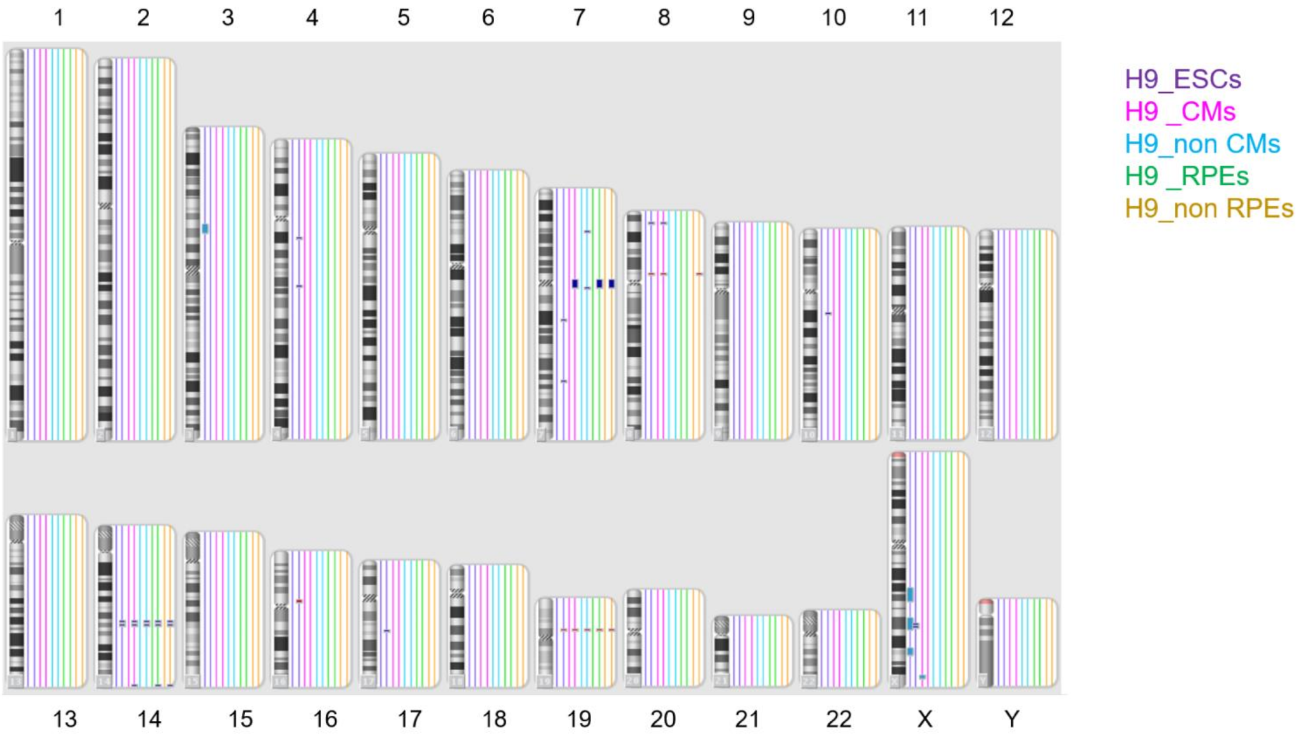

Fig. S3. A: Profile of CNVs from 16E84-iPSCs and their derivatives. B: Profile of CNVs from 16E85-iPSCs and their derivatives. C: Profile of CNVs from 16H12-iPSCs and their derivatives. D: Profile of CNVs from 15M38-iPSCs and their derivatives. E: Profile of CNVs from H9-iPSCs and their derivatives.

Fig.S4

A CNVs detected in Ff-WJ14s01-iPSCs and their derivatives

Copy number = 4 (red)

CNVs observed only in iPSCs

File	FfWJ1401_iPS	FfWJ1401_iPS
**CN State**	**3**	**0**
Type	Gain	Loss
Chromosome	7	Y
Cytoband Start	q31.32	q11.223
Size (kbp)	132.052	206.092
Marker Count	259	156
Gene Count	1	3
Genes	*CADPS2*	*DAZ3, DAZ4, DAZ2*

CNVs observed before and after differentiation

File	FfWJ1401_iPS	FfWJ1401_NSC	FfWJ1401_iPS	FfWJ1401_NSC	FfWJ1401_iPS	FfWJ1401_NSC	FfWJ1401_iPS	FfWJ1401_NSC	FfWJ1401_iPS	FfWJ1401_NSC	FfWJ1401_iPS	FfWJ1401_NSC	FfWJ1401_iPS	FfWJ1401_NSC
**CN State**	**3**	**3**	**3**	**3**	**3**	**3**	**3**	**3**	**4** (red)	**3**	**3**	**3**	**1**	**1**
Type	Gain	Gain	Gain	Gain	Gain	Gain	Gain	Gain	Gain	Gain	Gain	Gain	Loss	Loss
Chromosome	2	2	5	5	8	8	13	13	14	14	22	22	16	16
Cytoband Start	q23.3	q23.3	q35.3	q35.3	p11.22	p11.22	q12.12	q12.12	q32.33	q32.33	q11.22	q11.22	p11.2	p11.2
Size (kbp)	167.379	168.393	219.303	201.787	154.025	166.703	199.759	204.623	407.886	478.397	100.807	159.149	394.374	394.374
Marker Count	128	130	124	123	74	86	288	289	56	157	86	94	60	60
Gene Count	1	1	1	1	2	2	2	2	2	6	1	1	4	4
Genes	*RPRM*	*RPRM*	*ADAMTS2*	*ADAMTS2*	*ADAM5, ADAM3A*	*ADAM5, ADAM3A*	*SPATA13, MIR2276*	*SPATA13, MIR2276*	*KIAA0125, ADAM6*	*MIR4539, MIR4507, MIR4538, MIR4537, KIAA0125, ADAM6*	*MIR650*	*MIR650*	*TP53TG3B, TP53TG3, TP53TG3C, SLC6A10P*	*TP53TG3B, TP53TG3, TP53TG3C, SLC6A10P*

B CNVs detected in 1210B2-iPSCs and their derivatives

Copy number = 4

	CNVs observed only in iPSCs			CNVs newly acquired after differentiation		CNVs observed before and after differentiation			
File	1210B2_iPS	1210B2_iPS	1210B2_iPS	1210B2_NPC	1210B2_NPC	1210B2_iPS	1210B2_NPC	1210B2_iPS	1210B2_NPC
**CN State**	**3**	**3**	**2.2546268**	**3**	**3**	**1**	**1**	**1**	**1**
Type	Gain	Gain	GainMosaic	Gain	Gain	Loss	Loss	Loss	Loss
Chromosome	4	7	3	10	22	13	13	20	20
Cytoband Start	q13.1	q31.32	p14.2	q11.23	q11.22	q12.11	q12.11	p11.1	p11.1
Size (kbp)	135.294	571.687	6294.992	122.392	156.472	160.797	162.486	3956.559	3956.559
Marker Count	121	948	6539	72	90	182	186	56	56
Gene Count	2	4	32	3	1	1	1	7	7
Genes	*ADGRL3, ADGRL3-AS1*	*AASS, FEZF1, FEZF1-AS1, CADPS2*	*FHIT, PTPRG, PTPRG-AS1, C3orf14, FEZF2, CADPS, LINC00698, SYNPR, SYNPR-AS1, SNTN, C3orf49, THOC7, THOC7-AS1, ATXN7, PSMD6-AS2, PSMD6, PRICKLE2-AS1, LINC00994, PRICKLE2, PRICKLE2-AS2, PRICKLE2-AS3, ADAMTS9, ADAMTS9-AS1, ADAMTS9-AS2, MAGI1, MAGI1-AS1, SLC25A26, LRIG1, LOC105377143, KBTBD8, MIR4272, SUCLG2*	*PARG, TIMM23B, AGAP6*	*MIR650*	*LINC00442*	*LINC00442*	*FRG1CP, FRG1DP, FRG2EP, LOC105379477, LINC01598, MIR4477A, MIR4477B*	*FRG1CP, FRG1DP, FRG2EP, LOC105379477, LINC01598, MIR4477A, MIR4477B*

C CNVs detected in Ff-I01-iPSCs and their derivatives

Sample from transplant   Copy number = 4

	Ff-I01_ NSC p8	Ff-I01_ RPE p3	Ff-I01_ RPE+G1	Ff-I01_ RPE+G1	Ff-I01_ RPE+G1	Ff-I01_ RPE p3	Ff-I01_ NSC p8	Ff-I01_ RPE p3	Ff-I01_ RPE+G1	Ff-I01_ RPE+G1	Ff-I01_ NSC p8	Ff-I01_ NSC p8	Ff-I01_ RPE p3	Ff-I01_ RPE p3	Ff-I01_ RPE+G1	Ff-I01_ RPE+G1	Ff-I01_ NSC p8
**CN State**	**1**	**1**	**1**	**1**	**1**	**3**	**3**	**4**	**3**	**3**	**4**	**3**	**4**	**3**	**3**	**3**	**0**
Type	Loss	Loss	Loss	Loss	Loss	Gain	Gain	Gain	Gain	Gain	Gain	Gain	Gain	Gain	Gain	Gain	Loss
Chromosome	6	6	6	6	6	8	14	14	14	14	17	17	17	17	17	17	Y
Cytoband Start	q26	q26	q26	p25.3	q26	p11.22	q32.33	q32.33	q32.33	q32.33	q12	q12	q12	q12	q12	q12	q11.223
Genes	*PARK2*	*PARK2*	*PARK2*	*DUSP22*	*PARK2*	*ADAM5, ADAM3A*	*MIR4539, MIR4507, MIR4538, MIR4537, KIAA0125, ADAM6*	*KIAA0125, ADAM6*	*MIR4539, MIR4507, MIR4538, MIR4537, KIAA0125, ADAM6*	*MIR4539, MIR4507, MIR4538, MIR4537, KIAA0125, ADAM6*	*CCL4L1, CCL4L2, CCL4, TBC1D3H, TBC1D3I, TBC1D3G, LOC101060389, TBC1D3L, TBC1D3B, CCL3L3, CCL3L1, LOC440434, TBC1D3K, TBC1D3F*	*YWHAEP7, TBC1D3, TBC1D3K, TBC1D3H, TBC1D3I, LOC101060389, TBC1D3L*	*CCL4L1, CCL4L2, CCL4, TBC1D3H, TBC1D3I, TBC1D3G, LOC101060389, TBC1D3L, TBC1D3B, CCL3L3, CCL3L1, LOC440434, TBC1D3K, TBC1D3F*	*YWHAEP7*	*YWHAEP7, TBC1D3, TBC1D3K, TBC1D3H, TBC1D3I, LOC101060389, TBC1D3L, TBC1D3C, TBC1D3F, TBC1D3G, TBC1D3E, LOC440434*	*YWHAEP7, TBC1D3, TBC1D3K, TBC1D3H, TBC1D3I, LOC101060389, TBC1D3L, TBC1D3C, TBC1D3F, TBC1D3G, TBC1D3E, LOC440434*	*TTTY3, TTTY3B*

D CNVs detected in H9 cells and their derivatives

CNVs observed only in iPSCs

File	H9_ ESC	H9_ ESC	H9_ ESC	H9_ ESC	H9_ ESC	H9_ ESC	H9_ ESC	H9_ ESC	H9_ ESC	H9_ ESC	H9_ ESC	H9_ ESC
**CN State**	**2.2363653**	**3**	**3**	**3**	**3**	**1**	**3**	**2.2046766**	**2.2720542**	**3**	**3**	**2.3183916**
Type	GainMosaic	Gain	Gain	Gain	Gain	Loss	Gain	GainMosaic	GainMosaic	Gain	Gain	GainMosaic
Chromosome	3	4	4	7	7	16	17	X	X	X	X	X
Cytoband Start	p14.2	q13.1	q22.1	q21.11	q31.32	p11.2	q21.31	q21.31	q23	q23	q23	q26.1
Size (kbp)	5502.707	455.207	630.995	165.699	517.617	1412.952	576.398	9057.28	7652.548	824.269	321.172	4523.499
Marker Count	5738	392	616	284	704	112	80	5900	9526	382	360	10143
Gene Count	28	2	2	1	5	6	8	11	40	2	2	41
Genes	*PTPRG, PTPRG-AS1, C3orf14, FEZF2, CADPS, LINC00698, SYNPR, SYNPR-AS1, SNTN, C3orf49, THOC7, THOC7-AS1, ATXN7, PSMD6-AS2, PSMD6, PRICKLE2-AS1, LINC00994, PRICKLE2, PRICKLE2-AS2, PRICKLE2-AS3, ADAMTS9, ADAMTS9-AS1, ADAMTS9-AS2, MAGI1, MAGI1-AS1, SLC25A26, LRIG1, LOC105377143*	*ADGRL3, ADGRL3-AS1*	*LOC101929194, GRID2*	*SEMA3E*	*PTPRZ1, AASS, FEZF1, FEZF1-AS1, CADPS2*	*TP53TG3B, TP53TG3, TP53TG3C, SLC6A10P, LOC390705, ENPP7P13*	*KANSL1, KANSL1-AS1, LRRC37A, ARL17B, ARL17A, NSFP1, LRRC37A2, NSF*	*PABPC5-AS1, PABPC5, PCDH11X, MIR4454, NAP1L3, FAM133A, MIR548M, BRDTP1, DIAPH2, RPA4, DIAPH2-AS1*	*TMEM164, MIR652, MIR3978, AMMECR1, SNORD96B, RGAG1, TDGF1P3, CHRDL1, PAK3, CAPN6, DCX, LINC00890, ALG13, TRPC5, TRPC5OS, ZCCHC16, LHFPL1, AMOT, MIR4329, LOC101928437, XACT, HTR2C, SNORA35, MIR764, MIR1912, MIR1264, MIR1298, MIR1911, MIR448, IL13RA2, LRCH2, RBMXL3, LUZP4, PLS3-AS1, PLS3, DANT2, AGTR2, SLC6A14, CT83, LOC100126447*	*XACT, HTR2C*	*PLS3, DANT2*	*RBMX2, FAM45BP, ENOX2, LOC105373338, LINC01201, ARHGAP36, IGSF1, OR13H1, FIRRE, STK26, FRMD7, RAP2C, RAP2C-AS1, MBNL3, HS6ST2, HS6ST2-AS1, USP26, TFDP3, GPC4, GPC3, MIR363, MIR92A2, MIR19B2, MIR20B, MIR18B, MIR106A, CCDC160, PHF6, HPRT1, MIR450B, MIR450A1, MIR450A2, MIR542, MIR503HG, MIR503, MIR424, LINC00629, PLAC1, FAM122B, FAM122C, MOSPD1*

D CNVs detected in H9 cells and their derivatives

CNVs newly acquired after differentiation

File	H9_non CM	H9_non CM	H9_CM	H9_ RPE	H9_ non RPE	H9_CM
CN State	3	3	3	3	3	2.2094336
Type	Gain	Gain	Gain	Gain	Gain	GainMosaic
Chromosome	7	7	14	14	14	X
Cytoband Start	p15.2	q11.21	q32.33	q32.33	q32.33	q28
Size (kbp)	139.348	315.438	520.686	457.028	457.028	2139.135
Marker Count	154	78	169	140	140	7308
Gene Count	19	1	6	6	6	19
Genes	*HOXA1, HOTAIRM1, HOXA2, HOXA3, HOXA-AS2, HOXA4, HOXA-AS3, HOXA5, HOXA6, HOXA7, HOXA9, HOXA10-HOXA9, HOXA10-AS, MIR196B, HOXA10, HOXA11, HOXA11-AS, HOXA13, HOTTIP*	*ZNF733P*	*MIR4539, MIR4507, MIR4538, MIR4537, KIAA0125, ADAM6*	*MIR4539, MIR4507, MIR4538, MIR4537, KIAA0125, ADAM6*	*MIR4539, MIR4507, MIR4538, MIR4537, KIAA0125, ADAM6*	*AFF2, IDS, LINC00893, CXorf40A, LOC101928917, MAGEA9, MAGEA9B, HSFX2, HSFX1, TMEM185A, MAGEA11, LINC00850, MAGEA8-AS1, MAGEA8, CXorf40B, LINC00894, MIR2114, MAMLD1, MTM1*

D CNVs detected in H9 cells and their derivatives

CNVs observed before and after differentiation

File	H9_ ESC	H9_ CM	H9_ ESC	H9_ CM	H9_ non RPE	H9_ ESC	H9_ CM	H9_ non CM	H9_ RPE	H9_ non RPE	H9_ ESC	H9_ CM	H9_ non HCM	H9_ RPE	H9_ non RPE	H9_ ESC	H9_ CM	H9_ non CM	H9_ RPE	H9_ non RPE
**CN State**	**3**	**3**	**1**	**1**	**1**	**3**	**3**	**3**	**3**	**3**	**3**	**3**	**3**	**3**	**3**	**1**	**1**	**1**	**1**	**1**
Type	Gain	Gain	Loss	Loss	Loss	Gain	Gain	Gain	Gain	Gain	Gain	Gain	Gain	Gain	Gain	Loss	Loss	Loss	Loss	Loss
Chromosome	8	8	8	8	8	14	14	14	14	14	14	14	14	14	14	19	19	19	19	19
Cytoband Start	p23.1	p23.1	p11.22	p11.22	p11.22	q23.2	q23.2	q23.2	q23.2	q23.2	q23.3	q23.3	q23.3	q23.3	q23.3	p12	p12	p12	p12	p12
Size (kbp)	1104.225	1104.225	136.649	149.343	131.348	295.834	293.08	303.921	303.921	303.921	404.811	422.721	422.349	424.779	410.554	104.562	107.28	122.431	117.643	124.676
Marker Count	68	68	72	72	68	332	328	332	332	332	408	416	415	418	409	68	70	84	80	86
Gene Count	31	31	2	2	2	3	3	3	3	3	1	1	1	1	1	1	1	2	2	2
Genes	*LINC00965, FAM66B, DEFB109P1, DEFB109P1B, USP17L1, USP17L4, ZNF705G, DEFB4B, DEFB103B, DEFB103A, SPAG11B, DEFB104B, DEFB104A, DEFB106B, DEFB106A, DEFB105B, DEFB105A, DEFB107B, DEFB107A, PRR23D1, PRR23D2, FAM90A7P, FAM90A10P, SPAG11A, DEFB4A, ZNF705B, FAM66E, USP17L8, USP17L3, MIR548I3, FAM86B3P*	*LINC00965, FAM66B, DEFB109P1, DEFB109P1B, USP17L1, USP17L4, ZNF705G, DEFB4B, DEFB103B, DEFB103A, SPAG11B, DEFB104B, DEFB104A, DEFB106B, DEFB106A, DEFB105B, DEFB105A, DEFB107B, DEFB107A, PRR23D1, PRR23D2, FAM90A7P, FAM90A10P, SPAG11A, DEFB4A, ZNF705B, FAM66E, USP17L8, USP17L3, MIR548I3, FAM86B3P*	*ADAM5, ADAM3A*	*ADAM5, ADAM3A*	*ADAM5, ADAM3A*	*KCNH5, RHOJ, GPHB5*	*KCNH5, RHOJ, GPHB5*	*KCNH5, RHOJ, GPHB5*	*KCNH5, RHOJ, GPHB5*	*KCNH5, RHOJ, GPHB5*	*FUT8*	*FUT8*	*FUT8*	*FUT8*	*FUT8*	*ZNF826P*	*ZNF826P*	*ZNF826P, ZNF737*	*ZNF826P, ZNF737*	*ZNF826P, ZNF737*

E CNVs detected in 16E84 cells and their derivatives

CNVs observed only in iPSCs

File	16E84_ iPSC	16E84_ iPSC
**CN State**	**3**	**0**
Type	Gain	Loss
Chromosome	7	Y
Cytoband Start	q31.32	q11.223
Size (kbp)	125.818	295.853
Marker Count	232	60
Gene Count	3	2
Genes	*FEZF1, FEZF1-AS1, CADPS2*	*TTTY3, TTTY3B*

E CNVs detected in 16E84 cells and their derivatives

Copy number = 4

CNVs observed before and after differentiation

File	16E84_ iPSC	16E84_ CM	16E84_ non-CM	16E84_ iPSC	16E84_ RPE	16E84_ non-RPE	16E84_ CM	16E84_ non-CM	16E84_ iPSC	16E84_ RPE	16E84_ non-RPE	16E84_ CM	16E84_ non-CM	16E84_ iPSC	16E84_ RPE	16E84_ non-RPE	16E84_ non-RPE	16E84_ CM	16E84_ non-CM	16E84_ CM	16E84_ non-CM
**CN State**	**1**	**1**	**1**	**1**	**1**	**1**	**1**	**1**	**4**	**4**	**3**	**3**	**3**	**3**	**3**	**3**	**4**	**3**	**3**	**4**	**3**
Type	Loss	Loss	Loss	Loss	Loss	Loss	Loss	Loss	Gain	Gain	Gain	Gain	Gain	Gain	Gain	Gain	Gain	Gain	Gain	Gain	Gain
Chromosome	3	3	3	6	6	6	6	6	14	14	14	14	14	17	17	17	17	17	17	17	17
Cytoband Start	p14.2	p14.2	p14.2	q26	q26	q26	q26	q26	q32.33	q32.33	q32.33	q32.33	q32.33	q12	q12	q12	q12	q12	q12	q12	q12
Size (kbp)	326.226	336.324	336.324	149.529	149.529	147.433	147.433	147.433	407.886	407.886	485.71	576.567	485.71	205.038	205.016	205.038	367.77	205.016	205.016	367.77	367.77
Marker Count	430	434	434	194	194	192	192	192	56	56	164	180	164	106	104	106	64	104	104	64	64
Gene Count	1	1	1	1	1	1	1	1	2	2	6	6	6	7	7	7	14	7	7	14	14
Genes	*PTPRG*	*PTPRG*	*PTPRG*	*PARK2*	*PARK2*	*PARK2*	*PARK2*	*PARK2*	*KIAA0125, ADAM6*	*KIAA0125, ADAM6*	*MIR4539, MIR4507, MIR4538, MIR4537, KIAA0125, ADAM6*	*MIR4539, MIR4507, MIR4538, MIR4537, KIAA0125, ADAM6*	*MIR4539, MIR4507, MIR4538, MIR4537, KIAA0125, ADAM6*	*YWHAEP7, TBC1D3, TBC1D3K, TBC1D3H, TBC1D3I, LOC101060389, TBC1D3L*	*YWHAEP7, TBC1D3, TBC1D3K, TBC1D3H, TBC1D3I, LOC101060389, TBC1D3L*	*YWHAEP7, TBC1D3, TBC1D3K, TBC1D3H, TBC1D3I, LOC101060389, TBC1D3L*	*CCL4L1, CCL4L2, CCL4, TBC1D3H, TBC1D3I, LOC101060389, TBC1D3G, TBC1D3L, TBC1D3B, CCL3L3, CCL3L1, LOC440434, TBC1D3K, TBC1D3F*	*YWHAEP7, TBC1D3, TBC1D3K, TBC1D3H, TBC1D3I, LOC101060389, TBC1D3L*	*YWHAEP7, TBC1D3, TBC1D3K, TBC1D3H, TBC1D3I, LOC101060389, TBC1D3L*	*CCL4L1, CCL4L2, CCL4, TBC1D3H, TBC1D3I, LOC101060389, TBC1D3G, TBC1D3L, TBC1D3B, CCL3L3, CCL3L1, LOC440434, TBC1D3K, TBC1D3F*	*CCL4L1, CCL4L2, CCL4, TBC1D3H, TBC1D3I, LOC101060389, TBC1D3G, TBC1D3L, TBC1D3B, CCL3L3, CCL3L1, LOC440434, TBC1D3K, TBC1D3F*

F CNVs detected in 16E85-iPSCs and their derivatives

Copy number = 4

CNVs observed only in iPSCs

File	16E85_ iPS	16E85_ iPS
**CN State**	**3**	**3**
Type	Gain	Gain
Chromosome	4	7
Cytoband Start	q22.1	q31.32
Size (kbp)	140.734	131.253
Marker Count	144	208
Gene Count	1	3
Genes	*GRID2*	*FEZF1, FEZF1-AS1, CADPS2*

CNVs newly acquired after differentiation

16E85_ CM	16E85_ non-CM	16E85_ CM	16E85_ CM	16E85_ CM	16E85_ non-CM	16E85_ CM	16E85_ CM
**3**	**3**	**3**	**3**	**3**	**3**	**3**	**0**
Gain	Gain	Gain	Gain	Gain	Gain	Gain	Loss
1	1	6	6	7	7	17	Y
q21.3	q21.3	p22.2	p22.1	p15.2	p15.2	p13.1	q11.23
215.953	234.789	178.283	100.178	118.37	128.137	112.7	205.464
76	116	140	60	137	142	80	52
18	20	18	1	19	19	11	9
DCST1, LOC100505666, ADAM15, EFNA4, EFNA3, EFNA1, SLC50A1, DPM3, KRTCAP2, TRIM46, MUC1, MIR92B, THBS3, MTX1, GBAP1, GBA, FAM189B, SCAMP3	*DCST1, LOC100505666, ADAM15, EFNA4, EFNA3, EFNA1, SLC50A1, DPM3, KRTCAP2, TRIM46, MUC1, MIR92B, THBS3, MTX1, GBAP1, GBA, FAM189B, SCAMP3, CLK2, HCN3*	*HIST1H2BD, HIST1H2BE, HIST1H4D, HIST1H3D, HIST1H2AD, HIST1H2BF, HIST1H4E, HIST1H2BG, HIST1H2AE, HIST1H3E, HIST1H1D, HIST1H4F, HIST1H4G, HIST1H3F, HIST1H2BH, HIST1H3G, HIST1H2BI, HIST1H4H*	*ZNF184*	*HOXA1, HOTAIRM1, HOXA2, HOXA3, HOXA-AS2, HOXA4, HOXA-AS3, HOXA5, HOXA6, HOXA7, HOXA9, HOXA10-HOXA9, HOXA10-AS, MIR196B, HOXA10, HOXA11, HOXA11-AS, HOXA13, HOTTIP*	*HOXA1, HOTAIRM1, HOXA2, HOXA3, HOXA-AS2, HOXA4, HOXA-AS3, HOXA5, HOXA6, HOXA7, HOXA9, HOXA10-HOXA9, HOXA10-AS, MIR196B, HOXA10, HOXA11, HOXA11-AS, HOXA13, HOTTIP*	*HES7, PER1, MIR6883, VAMP2, TMEM107, SNORD118, MIR4521, BORCS6, AURKB, LINC00324, CTC1*	*DAZ3, DAZ4, DAZ2, BPY2C, BPY2B, BPY2, TTTY4, TTTY4B, TTTY4C*

F CNVs detected in 16E85-iPSCs and their derivatives

Copy number = 4

CNVs observed before and after differentiation

File	16E85_ iPS	16E85_ CM	16E85_ non-CM	16E85_ RPE	16E85_ non-RPE	16E85_ iPS	16E85_ RPE	16E85_ non-RPE	16E85_ non CM	16E85_ iPS	16E85_ non-CM
**CN State**	**1**	**1**	**1**	**1**	**1**	**3**	**3**	**3**	**4**	**3**	**4**
Type	Loss	Loss	Loss	Loss	Loss	Gain	Gain	Gain	Gain	Gain	Gain
Chromosome	6	6	6	6	6	14	14	14	14	17	17
Cytoband Start	q26	q26	q26	q26	q26	q32.33	q32.33	q32.33	q32.33	q12	q12
Size (kbp)	147.433	147.433	147.433	149.529	152.046	407.886	485.71	485.71	407.886	367.77	367.77
Marker Count	192	192	192	194	195	56	164	164	56	64	64
Gene Count	1	1	1	1	1	2	6	6	2	14	14
Genes	*PARK2*	*PARK2*	*PARK2*	*PARK2*	*PARK2*	*KIAA0125, ADAM6*	*MIR4539, MIR4507, MIR4538, MIR4537, KIAA0125, ADAM6*	*MIR4539, MIR4507, MIR4538, MIR4537, KIAA0125, ADAM6*	*KIAA0125, ADAM6*	*CCL4L1, CCL4L2, CCL4, TBC1D3H, TBC1D3I, LOC101060389, TBC1D3G, TBC1D3L, TBC1D3B, CCL3L3, CCL3L1, LOC440434, TBC1D3K, TBC1D3F*	*CCL4L1, CCL4L2, CCL4, TBC1D3H, TBC1D3I, LOC101060389, TBC1D3G, TBC1D3L, TBC1D3B, CCL3L3, CCL3L1, LOC440434, TBC1D3K, TBC1D3F*

File	16E85_ RPE	16E85_ non-RPE	16E85_ iPS	16E85_ CM	16E85_ non-CM	16E85_ RPE	16E85_ non-RPE	16E85_ iPS	16E85_ RPE	16E85_ non-RPE	16E85_ non-CM
**CN State**	**4**	**4**	**3**	**3**	**3**	**3**	**3**	**0**	**0**	**0**	**0**
Type	Gain	Gain	Gain	Gain	Gain	Gain	Gain	Loss	Loss	Loss	Loss
Chromosome	17	17	17	17	17	17	17	Y	Y	Y	Y
Cytoband Start	q12	q12	q12	q12	q12	q12	q12	q11.223	q11.223	q11.223	q11.223
Size (kbp)	367.77	367.77	205.016	205.016	205.038	205.016	410.448	296.203	219.036	295.853	296.107
Marker Count	64	64	104	104	106	104	110	64	168	60	62
Gene Count	14	14	7	7	7	7	12	2	3	2	2
Genes	*CCL4L1, CCL4L2, CCL4, TBC1D3H, TBC1D3I, LOC101060389, TBC1D3G, TBC1D3L, TBC1D3B, CCL3L3, CCL3L1, LOC440434, TBC1D3K, TBC1D3F*	*CCL4L1, CCL4L2, CCL4, TBC1D3H, TBC1D3I, LOC101060389, TBC1D3G, TBC1D3L, TBC1D3B, CCL3L3, CCL3L1, LOC440434, TBC1D3K, TBC1D3F*	*YWHAEP7, TBC1D3, TBC1D3K, TBC1D3H, TBC1D3I, LOC101060389, TBC1D3L*	*YWHAEP7, TBC1D3, TBC1D3K, TBC1D3H, TBC1D3I, LOC101060389, TBC1D3L*	*YWHAEP7, TBC1D3, TBC1D3K, TBC1D3H, TBC1D3I, LOC101060389, TBC1D3L*	*YWHAEP7, TBC1D3, TBC1D3K, TBC1D3H, TBC1D3I, LOC101060389, TBC1D3L*	*YWHAEP7, TBC1D3, TBC1D3K, TBC1D3H, TBC1D3I, LOC101060389, TBC1D3L, TBC1D3C, TBC1D3F, TBC1D3G, TBC1D3E, LOC440434*	*TTTY3, TTTY3B*	*DAZ3, DAZ4, DAZ2*	*TTTY3, TTTY3B*	*TTTY3, TTTY3B*

G CNVs detected in 16H12-iPSCs and their derivatives

Copy number = 4

CNVs observed only in iPSCs

File	16H12_ iPSC	16H12_ iPSC
**CN State**	**3**	**3**
Type	Gain	Gain
Chromosome	4	7
Cytoband Start	q13.1	q31.32
Size (kbp)	209.149	431.245
Marker Count	204	588
Gene Count	1	4
Genes	*ADGRL3*	*AASS, FEZF1, FEZF1-AS1, CADPS2*

CNVs newly acquired after differentiation

File	16H12_ non-CM	16H12_ nonCM	16H12_ RPE	16H12_ non-RPE
**CN State**	**3**	**3**	**3**	**3**
Type	Gain	Gain	Gain	Gain
Chromosome	8	14	14	14
Cytoband Start	p11.22	q32.33	q32.33	q32.33
Size (kbp)	148.275	619.982	608.617	601.304
Marker Count	78	200	201	194
Gene Count	2	7	7	7
Genes	*ADAM5, ADAM3A*	*MIR4539, MIR4507, MIR4538, MIR4537, KIAA0125, ADAM6, LINC00226*	*MIR4539, MIR4507, MIR4538, MIR4537, KIAA0125, ADAM6, LINC00226*	*MIR4539, MIR4507, MIR4538, MIR4537, KIAA0125, ADAM6, LINC00226*

CNVs observed before and after differentiation

File	16H12_ iPSC	16H12_ RPE	16H12_ non-RPE	16H12_ iPSC	16H12_ non-CM	16H12_ RPE	16H12_ non-RPE	16H12_ iPSC	16H12_ non-CM	16H12_ non-RPE	16H12_ iPSC	16H12_ non-CM	16H12_ RPE
**CN State**	**1**	**1**	**1**	**3**	**3**	**3**	**3**	**3**	**3**	**4**	**1**	**1**	**1**
Type	Loss	Loss	Loss	Gain	Gain	Gain	Gain	Gain	Gain	Gain	Loss	Loss	Loss
Chromosome	1	1	1	15	15	15	15	17	17	17	19	19	19
Cytoband Start	q44	q44	q44	q22.2	q22.2	q22.2	q22.2	q12	q12	q12	p12	p12	p12
Size (kbp)	105.85	105.85	105.85	136.79	132.175	128.361	127.352	367.77	369.636	367.77	127.394	127.394	120.184
Marker Count	56	56	56	184	168	166	162	64	68	64	88	88	84
Gene Count	4	4	4	1	1	1	1	14	14	14	2	2	1
Genes	*OR2T29, OR2T34, OR2T10, OR2T11*	*OR2T29, OR2T34, OR2T10, OR2T11*	*OR2T29, OR2T34, OR2T10, OR2T11*	*RORA*	*RORA*	*RORA*	*RORA*	*CCL4L1, CCL4L2, CCL4, TBC1D3H, TBC1D3I, LOC101060389, TBC1D3G, TBC1D3L, TBC1D3B, CCL3L3, CCL3L1, LOC440434, TBC1D3K, TBC1D3F*	*CCL4L1, CCL4L2, CCL4, TBC1D3H, TBC1D3I, LOC101060389, TBC1D3G, TBC1D3L, TBC1D3B, CCL3L3, CCL3L1, LOC440434, TBC1D3K, TBC1D3F*	*CCL4L1, CCL4L2, CCL4, TBC1D3H, TBC1D3I, LOC101060389, TBC1D3G, TBC1D3L, TBC1D3B, CCL3L3, CCL3L1, LOC440434, TBC1D3K, TBC1D3F*	*ZNF826P, ZNF737*	*ZNF826P, ZNF737*	*ZNF826P*

H CNVs detected in 15M38-iPSCs and their derivatives

CNVs observed only in iPSCs

File	15M38_ iPSC	15M38_ iPSC	15M38_ iPSC	15M38_ iPSC	15M38_ iPSC	15M38_ iPSC	15M38_ iPSC
**CN State**	**2.2260864**	**3**	**3**	**3**	**3**	**1.7444303**	**1**
Type	GainMosaic	Gain	Gain	Gain	Gain	LossMosaic	Loss
Chromosome	3	3	4	4	7	12	16
Cytoband Start	p14.2	p14.1	q13.1	q22.1	q31.32	q24.32	p11.2
Size (kbp)	4599.826	126.4	269.475	117.186	265	4727.405	389.539
Marker Count	4954	60	268	104	498	4892	52
Gene Count	27	1	1	1	3	30	4
Genes	*PTPRG, PTPRG-AS1, C3orf14, FEZF2, CADPS, LINC00698, SYNPR, SYNPR-AS1, SNTN, C3orf49, THOC7, THOC7-AS1, ATXN7, PSMD6-AS2, PSMD6, PRICKLE2-AS1, LINC00994, PRICKLE2, PRICKLE2-AS2, PRICKLE2-AS3, ADAMTS9, ADAMTS9-AS1, ADAMTS9-AS2, MAGI1, MAGI1-AS1, SLC25A26, LRIG1*	*SLC25A26*	*ADGRL3*	*GRID2*	*FEZF1, FEZF1-AS1, CADPS2*	*LINC00939, LOC101927464, LOC100128554, LOC100996671, LINC00944, LINC00943, LOC440117, LOC101927592, LOC101927616, LOC101927637, LOC105370068, FLJ37505, LINC00508, LINC00507, CRAT8, LOC100996679, LOC101927694, MIR4419B, TMEM132C, MIR3612, SLC15A4, GLT1D1, TMEM132D, LOC283352, LOC101927735, LOC100190940, FZD10-AS1, FZD10, PIWIL1, RIMBP2*	*TP53TG3B, TP53TG3, TP53TG3C, SLC6A10P*

H CNVs detected in 15M38-iPSCs and their derivatives

Sample from transplant (yellow) Copy number = 4 (red)

CNVs observed only in iPSCs

File	15M38_ non-RPE	15M38_ non-HCM + G1	15M38_ non-CM	15M38_ non-CM	15M38_ non-HCM + G1	15M38_ RPE + G1	15M38_ RPE + G1	15M38_ non-CM	15M38_ RPE + G1	15M38_ RPE + G1
**CN State**	**1**	**1**	**3**	**3**	**1**	**1**	**1**	**3**	**3**	**0**
Type	Loss	Loss	Gain	Gain	Loss	Loss	Loss	Gain	Gain	Loss
Chromosome	4	6	8	11	11	11	12	22	22	Y
Cytoband Start	q13.2	q21	p11.22	q11	q23.2	q11	q23.3	q12.3	q11.22	q11.223
Size (kbp)	106.075	220.037	167.46	141.18	227.895	159.493	371.55	141.39	176.119	295.853
Marker Count	52	248	88	160	224	82	380	152	96	60
Gene Count	1	4	2	5	3	1	4	2	1	2
Genes	*UGT2B15*	*FLJ34503, HDAC2, LOC101927768, HS3ST5*	*ADAM5, ADAM3A*	*OR4C16, OR4C11, OR4P4, OR4S2, OR4C6*	*REXO2, NXPE1, NXPE4*	*TRIM48*	*TCP11L2, POLR3B, LOC100287944, RFX4*	*ISX, LINC01399*	*MIR650*	*TTTY3B, TTTY3*

H CNVs detected in 15M38-iPSCs and their derivatives

Sample from transplant | Copy number = 4

CNVs observed before and after differentiation

File	15M38_ iPSC	15M38_ non-CM	15M38_ non HCM + G1	15M38_ RPE	15M38_ RPE + G1	15M38_ non-RPE	15M38_ non-RPE + G1
**CN State**	**3**	**4**	**3**	**3**	**3**	**3**	**3**
Type	Gain	Gain	Gain	Gain	Gain	Gain	Gain
Chromosome	5	5	5	5	5	5	5
Cytoband Start	q35.3	q35.3	q35.3	q35.3	q35.3	q35.3	q35.3
Size (kbp)	180.293	176.696	219.303	219.303	180.377	201.787	194.182
Marker Count	111	109	124	124	112	123	115
Gene Count	1	1	1	1	1	1	1
Genes	*ADAMTS2*	*ADAMTS2*	*ADAMTS2*	*ADAMTS2*	*ADAMTS2*	*ADAMTS2*	*ADAMTS2*

File	15M38_ iPSC	15M38_ non-CM	15M38_ non HCM + G1	15M38_ RPE	15M38_ RPE + G1	15M38_ non-RPE	15M38_ non-RPE + G1
**CN State**	**3**	**3**	**3**	**3**	**3**	**3**	**3**
Type	Gain	Gain	Gain	Gain	Gain	Gain	Gain
Chromosome	13	13	13	13	13	13	13
Cytoband Start	q12.12	q12.12	q12.12	q12.12	q12.12	q12.12	q12.12
Size (kbp)	199.759	199.759	207.491	199.759	207.617	202.627	202.627
Marker Count	288	288	292	288	293	291	291
Gene Count	2	2	2	2	2	2	2
Genes	*SPATA13, MIR2276*	*SPATA13, MIR2276*	*SPATA13, MIR2276*	*SPATA13, MIR2276*	*SPATA13, MIR2276*	*SPATA13, MIR2276*	*SPATA13, MIR2276*

File	15M38_ iPSC	15M38_ non-CM	15M38_ non-HCM + G1	15M38_ RPE	15M38_ RPE + G1	15M38_ non-RPE	15M38_ non-RPE + G1
**CN State**	4	3	4	4	4	3	3
Type	Gain	Gain	Gain	Gain	Gain	Gain	Gain
Chromosome	14	14	14	14	14	14	14
Cytoband Start	q32.33	q32.33	q32.33	q32.33	q32.33	q32.33	q32.33
Size (kbp)	407.886	485.71	407.886	407.886	407.886	478.353	509.742
Marker Count	56	164	56	56	56	156	177
Gene Count	2	6	2	2	2	6	7
Genes	*KIAA0125, ADAM6*	*MIR4539, MIR4507, MIR4538, MIR4537, KIAA0125, ADAM6*	*KIAA0125, ADAM6*	*KIAA0125, ADAM6*	*KIAA0125, ADAM6*	*MIR4539, MIR4507, MIR4538, MIR4537, KIAA0125, ADAM6*	*MIR4539, MIR4507, MIR4538, MIR4537, KIAA0125, ADAM6, LINC00226*

Fig. S4. A: CNVs detected in Ff-WJ14s01-iPSCs and their derivatives. B: CNVs detected in 1210B2-iPSCs and their derivatives. C: CNVs detected in Ff-I01-iPSCs and their derivatives. D: CNVs detected in H9-ESCs and their derivatives. E: CNVs detected in 16E84-iPSCs and their derivatives. F: CNVs detected in 16E85-iPSCs and their derivatives. G: CNVs detected in 16H12-iPSCs and their derivatives. H: CNVs detected in 15M38-iPSCs and their derivatives.

Fig.S5.

14q32.33

Sample name	Probe ID	Copies/30 ng DNA		CN
		Target	reference	
16E84 iPSC	4868	60200	13740	8.77
16E84 RPEs	4868	26280	4340	12.1
16E84 non-RPEs	4868	26520	4300	12.3
16E84 CMs	4868	26940	3960	13.6
16E84 non-CMs	4868	30380	4180	14.5
16E85 iPSC	4868	77200	17960	8.6
16E85 RPEs	4868	30220	5060	11.96
16E85 non-RPEs	4868	19980	3100	12.9
16E85 CMs	4868	50000	14240	7.04
16E85 non-CMs	4868	48500	12980	7.47
16H12 iPSC	4868	10260	8480	2.42
16H12 RPEs	4868	5520	3740	2.95
16H12 non-RPEs	4868	4740	3180	2.97
16H12 non-CMs	4868	12720	11780	2.16
15M38 iPSC	4868	35040	13680	5.12
15M38 RPEs	4868	10800	2800	7.69
15M38 non-RPEs	4868	10640	3100	6.87
15M38 CMs	4868	30400	7660	7.94
H9 ESC	4868	12200	14320	1.7
H9 RPEs	4868	2840	3100	1.83
H9 non-RPEs	4868	3180	3480	1.83
H9 CMs	4868	7340	8120	1.8
H9 non-CMs	4868	7740	15700	0.986

17q12

Sample name	Probe ID	Copies/30 ng DNA		CN
		Target	reference	
16E84 iPSC	8378	20840	12280	3.4
16E84 RPEs	8378	8220	4160	3.96
16E84 non-RPEs	8378	7660	3920	3.91
16E84 CMs	8378	8340	4180	3.99
16E84 non-CMs	8378	8520	4180	4.08
16E85 iPSC	8378	29200	17440	3.35
16E85 RPEs	8378	9020	4700	3.85
16E85 non-RPEs	8378	5760	2940	3.91
16E85 CMs	8378	16100	14560	2.21
16E85 non-CMs	8378	15140	13520	2.24
16H12 iPSC	8378	14480	8240	3.52
16H12 RPEs	8378	7640	3920	3.9
16H12 non-RPEs	8378	6500	3220	4.03
16H12 non-CMs	8378	17480	12040	2.9
15M38 iPSC	8378	12540	13780	1.82
15M38 RPEs	8378	3100	2800	2.21
15M38 non-RPEs	8378	2860	3120	1.83
15M38 CMs	8378	8320	8280	2.01
H9 ESC	8378	19740	14820	2.66
H9 RPEs	8378	4620	3180	2.89
H9 non-RPEs	8378	5120	3660	2.8
H9 CMs	8378	10520	8060	2.61
H9 non-CMs	8378	11680	15340	1.52

Cytoband	Taqman Probe
14q32.33	Hs03124868_cn
17q12	Hs03958378_cn

Fig. S5. CNs detected at 14q32.33 or 17q12 by ddPCR. CN: copy number. The ribonuclease P RNA component *H1* gene (*RPPH1*) on Chr. 14q11.2 was used as a stable control for diploid copies (two copy control, CN = 2). The target locus in 14q32.33 was Chr.14:106260714, and that in 17q12 was Chr.17:36120285 on GRCh38. The amplicon lengths were approximately 100 bp. Samples with a copy number greater than 3 are highlighted in yellow.
